# Supplementary material for: Core-dependent post-translational modifications guide the biosynthesis of a new class of hypermodified peptides
Source: Nat Commun. 2023 Nov 25;14:7734. doi: 10.1038/s41467-023-43604-5 (PMC10676384; doi:10.1038/s41467-023-43604-5)

Supplementary Information

Core-Dependent Post-translational Modifications Guide the Biosynthesis of a New Class of Hypermodified Peptides

Zeng-Fei Pei[1], Lingyang Zhu[2], and Satish K. Nair[1,3,4]*

[1]Department of Biochemistry, University of Illinois at Urbana-Champaign, Roger Adams Laboratory, 600 S. Mathews Ave, Urbana, IL 61801, USA

[2]School of Chemical Sciences, NMR Laboratory, University of Illinois at Urbana-Champaign, Urbana, IL 61801, USA

[3]Carl R. Woese Institute for Genomic Biology, University of Illinois at Urbana-Champaign, 1206 W. Gregory Drive, Urbana, IL 61801, USA

[4]Center for Biophysics and Computational Biology, University of Illinois at Urbana-Champaign, Roger Adams Laboratory, 600 S. Mathews Ave., Urbana, IL 61801, USA

Table of Contents

Supplementary Figure 1: Representative dehydroamino acid and azol(in)e containing RiPPs……. S4

Supplementary Figure 2: Mechanism of dehydro amino acid and azol(in)e formation in RiPPs…..S5

Supplementary Figure 3: Classification of LanM based on the biosynthetic gene organization……S6

Supplementary Figure 4: Multiple sequence alignment and phylogenetic tree analysis……………S8

Supplementary Figure 5: Structural comparison of predicted CcaM and CylM……………….......S9

Supplementary Figure 6. Sequence alignment of CcaD and radical sam epimerase PoyD..…..... .S10

Supplementary Figure 7: SDS-PAGE analysis of purified proteins used in this study………..…...S11

Supplementary Figure 8: In vitro removal of leader peptide by CcaF…………………...…...…….S12

Supplementary Figure 9: In vitro CcaF assay of the synthetic 10-mer peptide…………………….S13

Supplementary Figure 10: MALDI-TOF mass spectra of cCcaA^{BCH}………………....……….…S14

Supplementary Figure 11: NMR spectra of cCcaA^{BCH} in 9:1 (v/v) H_2O/D_2O at 25 °C…………….S17

Supplementary Figure 12: MALDI-TOF mass spectra of carnazolamide……………………..…...…S18

Supplementary Figure 13: NMR spectra of carnazolamide in CD_3OH at 25 °C…………………...S20

Supplementary Figure 14: HPLC Marfey's analysis of cCcaA^{BCH} and cCcaA^{BCDH} lysates……….S22

Supplementary Figure 15: Marfey's assay of carnazolamide hydrolysate…………………………S23

Supplementary Figure 16: In vitro assay of full length CcaA with CcaM…………………………S24

Supplementary Figure 17: MALDI-TOF MS/MS of tri, hexa, hepta dehydrated peptide and carnazolamide……………………………………………………………………………..S25

Supplementary Figure 18: MALDI-TOF MS/MS of cCcaA^{BCH} truncants and CcaM catalyzed products…………………………………………………………………………………... S29

Supplementary Figure 19: MALDI-TOF mass spectra of cCcaA(T7A)^{BCH}……………………….S30

Supplementary Figure 20: Proposed dehydration mechanism of CcaM……………………..….S31

Supplementary Figure 21: Representative BGCs containing a novel LanM$_b$C and a YcaO……….S32

Supplementary Table 1: Plasmids constructed in this study……………………………………..S33

Supplementary Table 2: Combination of plasmids for heterologous expression in *E. coli*………..S34

Supplementary Table 3: Primers used in this study………………………………………………..S35

Supplementary Table 4: ^{1}H chemical shifts of cCcaA^{BCH} in 9:1 (v/v) H_2O/D_2O at 25 °C………...S36

Supplementary Table 5: [1]H chemical shifts of carnazolamide in CD_3OH at 25 °C……………….S37

Supplementary Table 6: Biological assay of carnazolamide…………………...……….………….S38

References……………………………………………………………………………………..S39

Uncropped protein gel………………………………………………………………………..S40

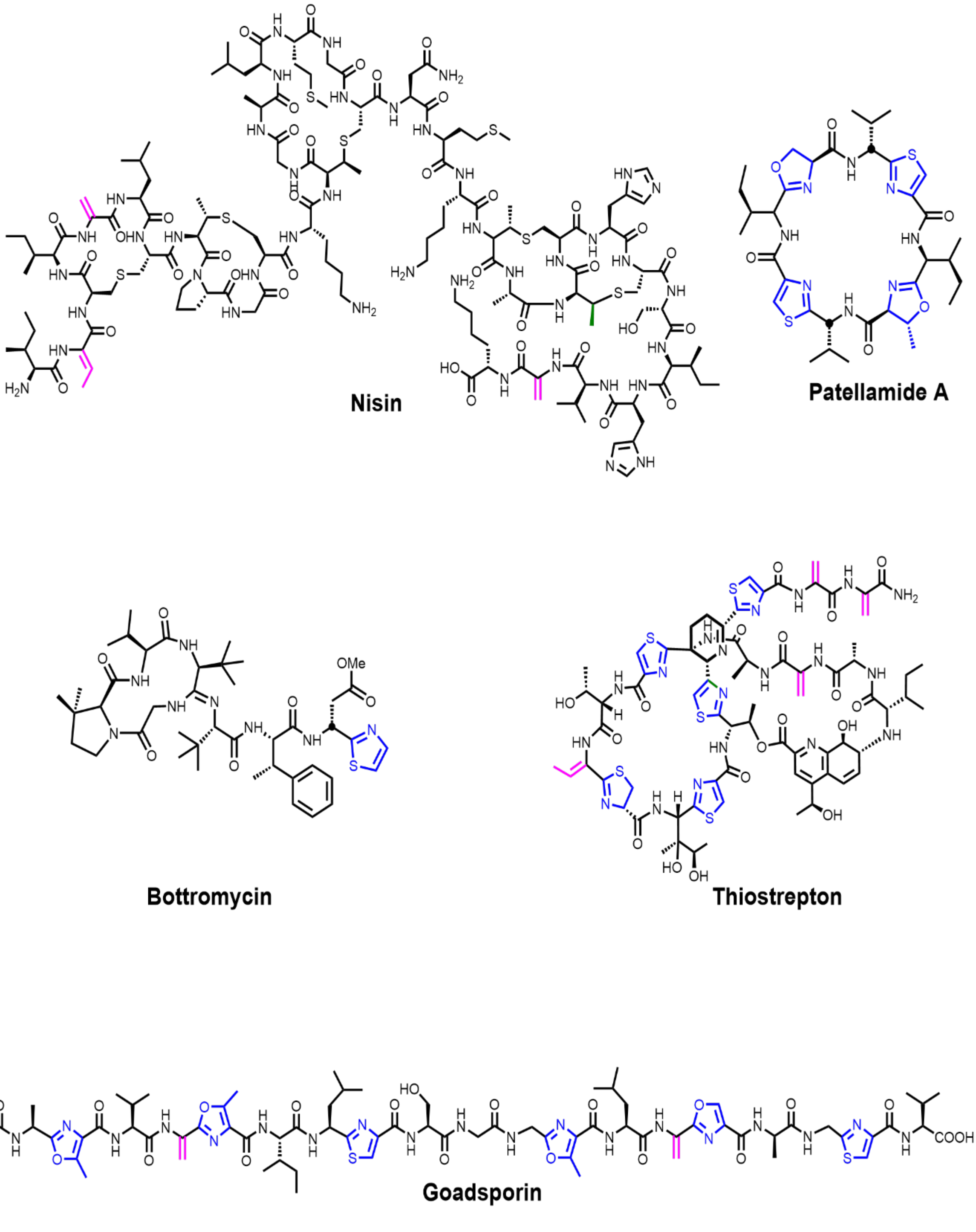

Supplementary Figure 1. Representative dehydroamino acid and azol(in)e containing RiPPs.

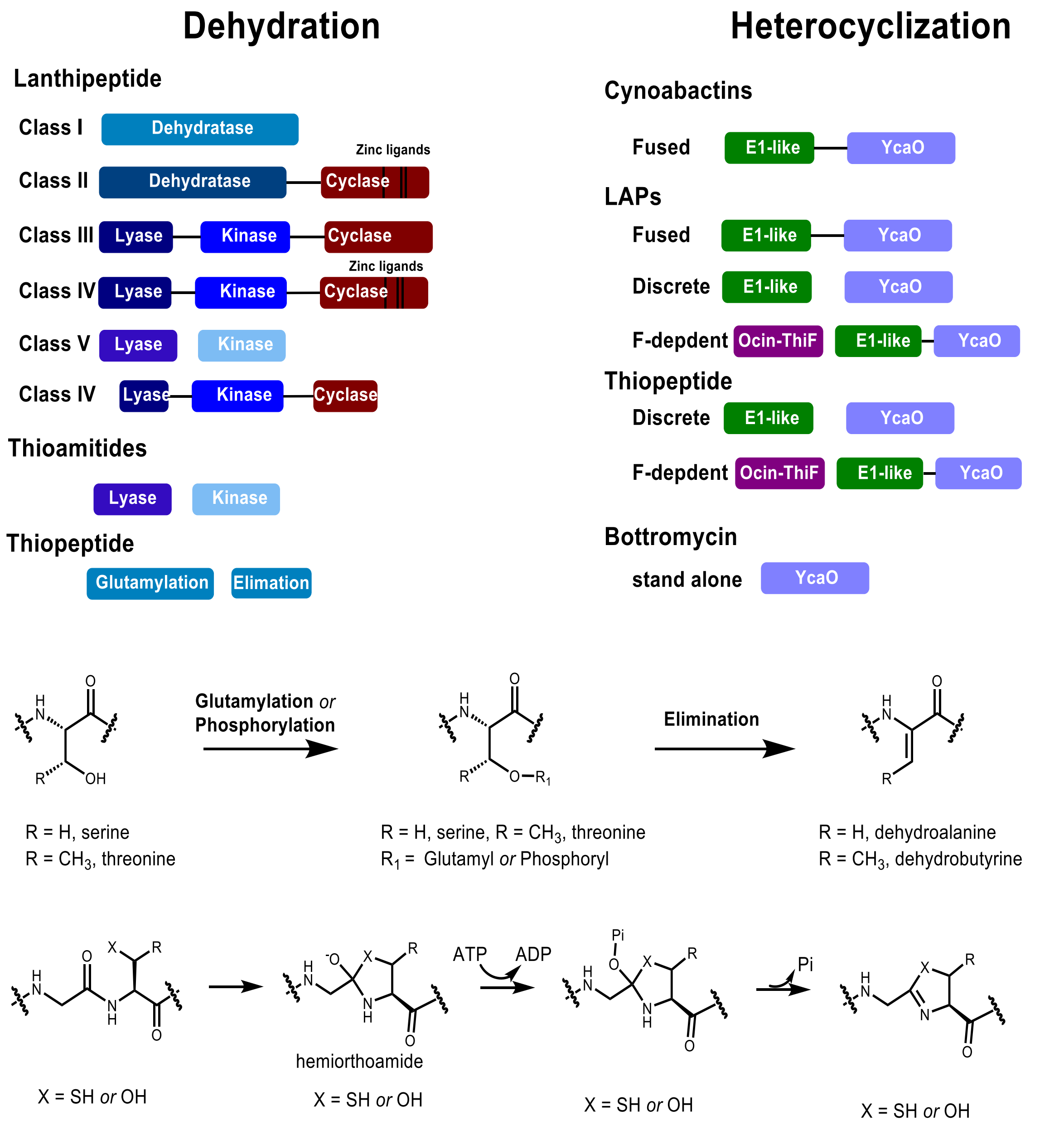

Supplementary Figure 2. Biosynthetic origin and mechanism of dehydro amino acid and azol(in)e in RiPPs.

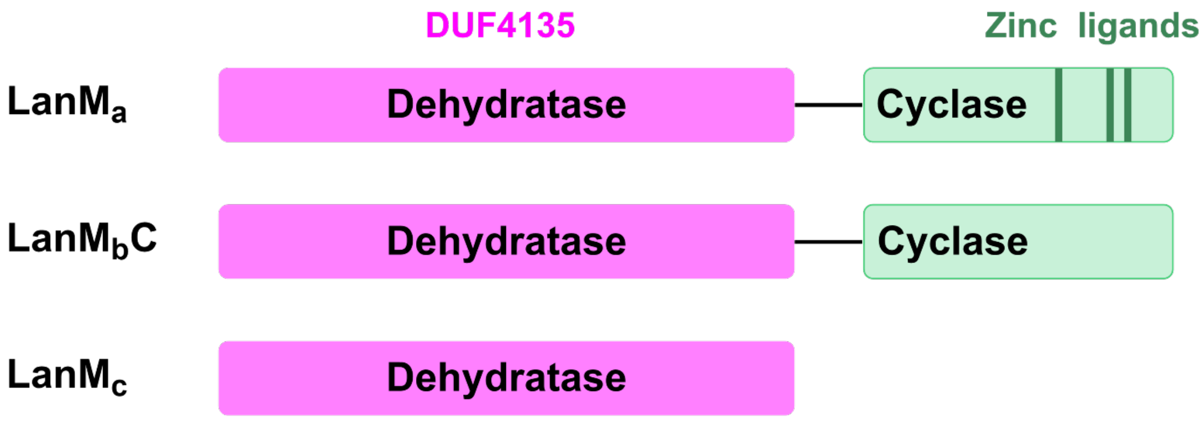

Supplementary Figure 3. Classification of LanM based on the biosynthetic gene organization.

a

```
                                                   **
CylM            YDDNIS----NIDINKTI-EYK-------NKDCLCHGNAGTLEGLIQLAK--------KD
A0A2N3KQU4      AGLL-----ESRQLRPLVIQSAQAKSKFFDDSISCLDLLVCCRLALALFKQ---------
A0A5R9M7I1      AVSLGTGAVHPGDLAWLTPPVT----AAEIAALGTRELVAAAGEALTSAEAGLLHTLDDD
A0A6B2VR55      AVSLGTGAVHPGDLAGLTPPVT----AAEIAALGTRELVAAAGEALTSAEAGLLHTLDDD
A0A6B2WA28      AVSLGTGAVHPGDLAGLTPPVT----AAEIAALGTRELVAAAGEALTSAEAGLLHTLDDD
A0A1I2LQC0      AVSLGAAALDSRQLGLLAAPVG----LAQVAGLGTAELVATAGEALTAAELGLVHSLPEG
A0A066YQS6      ------------------------------------------------------------
A0A1Q5EUS0      AVSLGADAVDGADLGALAPPVT----GPELAALTGRALLSAATEALTAAEAGLVHTLPEA
A0A7H8KY55      AVSLGADAVDQADLGALAPPVT----GPELAALTGRALLSAATEALTAAEAGLVHTLPED
E4NFR7          AVSLGADAVDQDDLGVLAPPVT----GPELAALTGRALLSAATEALTAAEAGLVHTLPEE
A0A419YLJ0      AVSLGADAVDQADLGALAPPVT----GPELAALTGRALLSAATEALTAAEAGLVHTLPEG
A0A327T358      AVSLGADAVDQADLGALAPPVT----GPELAALTGRALLSAATEALTAAEAGLVHTLPED
A0A3N4S9A4      AVSLGADAVDGADLGALAPPVT----GPELAALTGRALLAAATEALTAAEAGLVHTLPED
A0A8G1X9V3      AVSLGADAVDGADLGALAPPVT----GPELAALTGRALLAAATEALTAAEAGLVHTLPED
A0A561EXY4      AVSLGAGAVNPADLGELAPPVG----ESQILGLSTRALVATAGEALTAAEAGLLHTLPEG
A0A2A3I1T1      AVSLGAGSVDPAELGALTPPVG----AAGIAGRTTRDLVATAGEALTAAEAGLLHTVPEG
A0A0Q8PMZ3      AVALGAGIVDPAELAALTPPVS----ERQITGRSTRDLVATAGEALTAAEAGLLHTVPDP
A0A7W7SDA2      AVALGAGIVDPAELAALTPPVS----ARQISGRSTRDLVATAGEALTAAEAGLLHTVPEA
A0A097CRM5      ------------------------------------------------------------
F4FHE1          AAALGRAR--P-----AQAQTP----T-PAGSPSSVDLLDRIELGLAAWR----------
A0A1G9HJZ5      DHLLTGDV--SAELRERVARQC----A-PRARRSCERLVVDAELALHTAR----------
A0A4U3MMZ2      AHALTGEV--PADLRDHVARHC----A-PTPARSCAALVVDAEMAIHTAR----------
A0A8J3W1S2      AHLLTGEV--PGGLRERVARHC----A-PDPGRSCVRLVVDAEVALHTAR----------
A0A1H6EW28      AHLLTGEV--PGDLRERVARYC----A-ADAARSCARLVVDTEVALHTAR----------
A0A7W9LCK1      AHLLTGEV--PGDLRERVARYC----A-PDAARSCARLVVDAEVALHTAR----------
A0A2D8UIJ3      LELN-K---NNKSIRSYYDTFL----TKPPKKLRTTQLIEYADLSLNLYEI---------
A0A2E6EXT3      LELN-K---NNKSIRSYYDTFL----TKPPKKLRTTQLIEYADLSLNLYEI---------
A0A0Q4EB58      RY--------DQNSADVYAECL----SRDIRQLTVQQLIDYAELALNLSDI---------
A0A1I4VP56      RY--------DQKSADAYKTCL----SRDVGQLTTQQLIDYAELALNLSEI---------
A0A1M5H7Z5      RY--------DQNSADVYAARL----SRDIRQLTVQQLIDHAELALNLSDI---------
A0A1N7HZ13      RY--------DQNSADVYQAFL----SRDTRQLTTQQLIDYAELALNLSDI---------
CcaM            RY--------DQNSANVYQAFL----SRDIRQLTVQQLIDYAELALNLSDI---------
A0A376DRU0      RY--------DQNSANVYQAFL----SRDIRQLTVQQLIDYAELALNLSDI---------
A0A1E2WG51      IQSTRI---NAEQVLRKIDEYL----AVERLATDTVGLLDDLEVAITAFQV---------
A0A0K1EEA4      DELGVR---SLPELFARVDAYL----DVDPGSLGSRRLLGALELALVAYRR---------
A0A848LRJ3      ADGTAH---PALP--TSIRRVL-------RRAATASTSLEAVELALTAGRE---------
A0A2T4V8J4      G--------QGDGLRARVLHAL----EGAAS-GSTSARLDGLELALVADSS---------
A0A1L9AYM3      AREQAR---PGDSLRPRVLRAL----EEARAASSVAVQLDAIELALDAART---------
A0A250IW45      AREQER---PDGSLRPRVLRAL----EEARAASSVAVQLDAIELALDAAQT---------
S9PA60          AREQGQ---PGGSLRPRVLRAL----EEAHAASSVAVQLDAIELALDAAQT---------
```

b

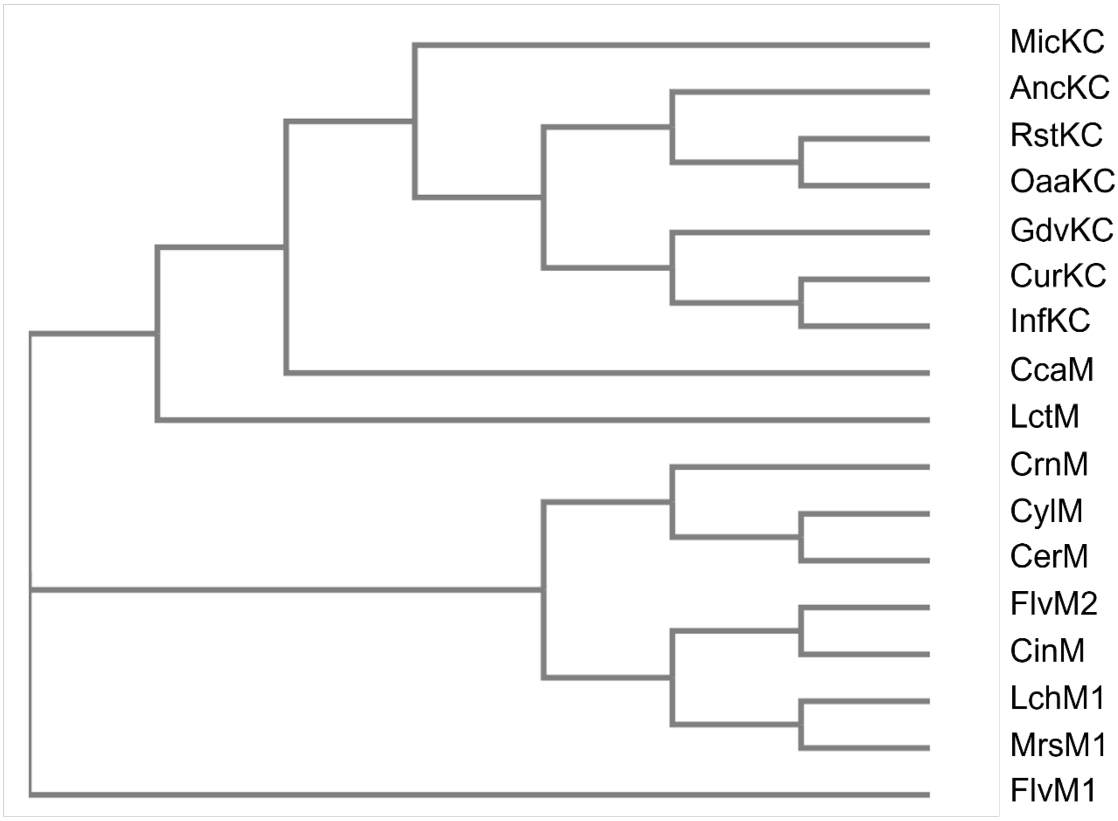

Supplementary Figure 4. Multiple sequence alignment and phylogenetic tree analysis. (**a**) Multiple sequence alignment of canonical zinc-binding CylM[1] with sequences of representative metal-independent LanM$_b$Cs. The asterisks designate the two conserved zinc binding sites in the cyclase domain of CylM (C911/H912). (**b**) Simple phylogenetic tree analysis of CcaM cyclase domain and cyclase domain of characterized conical Class II lanthipeptide synthetase LanM and class III lanthipeptide synthetase LanKC, it shows that CcaM cyclase domain is closer to that of LanKC.

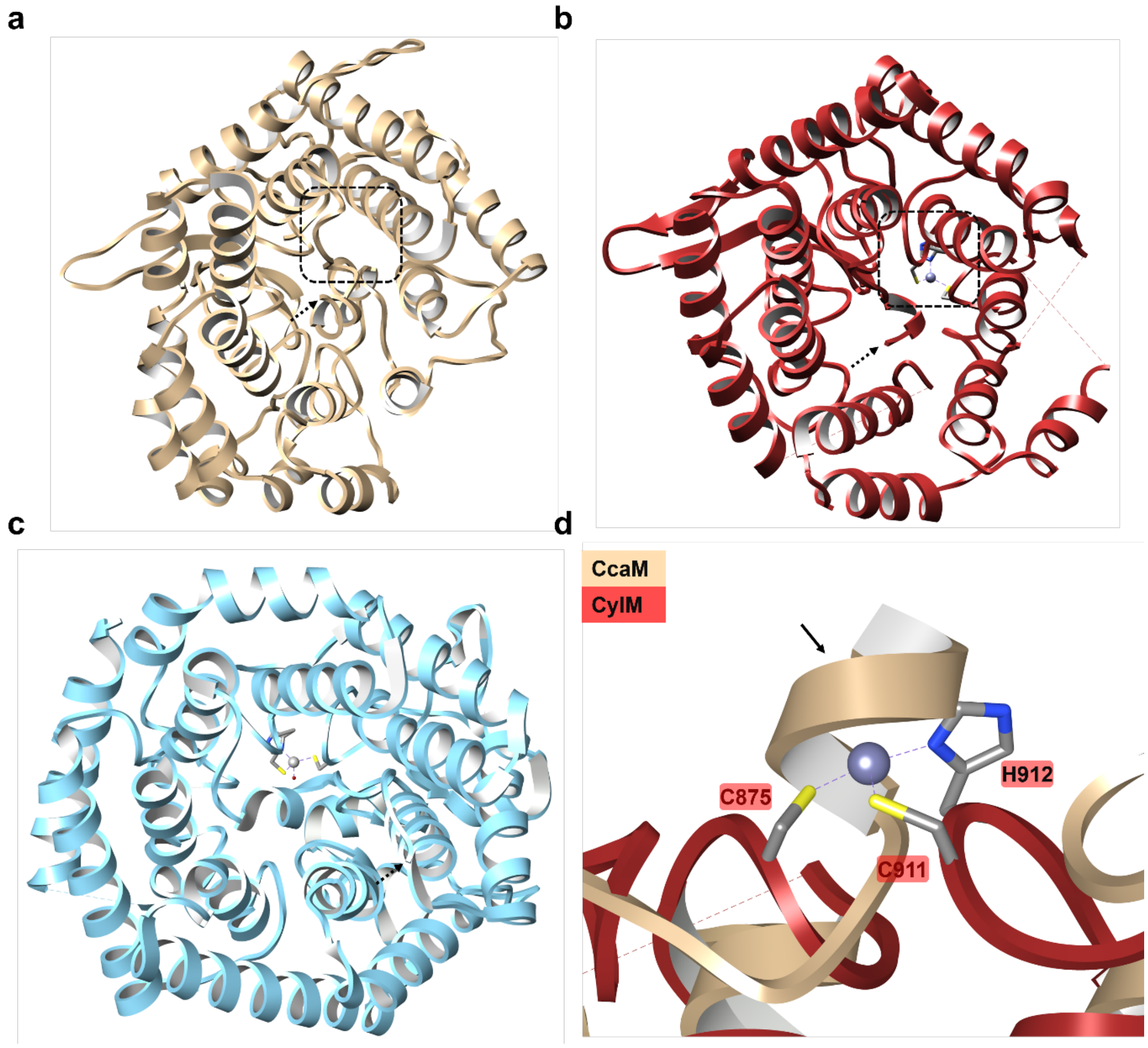

Supplementary Figure 5. Structural comparison of the cyclase domain of CcaM (predicted by AlphaFold[3]), CylM[1] (5DZT) and NisC[2] (2G02). Overall view of the cyclase domains CcaM (predicted) (**a**), CylM (**b**) and NisC (**c**). The dash arrow points to the C termini of the protein. Box indicates that the zoom-in region of CcaM and CylM. (**d**) Superposition of the model structure of CcaM with that of CylM shows that the zinc binding residues are not conserved in CcaM, and the conserved zinc binding pocket is occupied by a helix (pointed by an arrow) in the predicted CcaM structure.

```
CcaD      ----MQKSLSYRNSYHDNLHRYAGLKRFFERWSADPDFKQDLINNTTETLEKHAIPVTPD   56
PoyD      MNLQSIDSQAVRSKVDAEYPSAAHVKRFLEVWCSGLYKKEDLLTRPQEILDAHHVAIDPS   60
                .* : *.. . :    * :***:* *.:.   *:**:..  * *: * : : *.

CcaD      DIKYMIDERITE--------LPQPVQTMWEISKSKQDLIQSFYLSENLPRDRGMLAWRNR   108
PoyD      LISVLFESKFLRGKSGKFDLLPPQFDAFRDFMMTKIQW-RQQIRTGSAPADPIFREFRER   119
           *. :::.:: .        **  .::: ::  :* :  :.   : . * *  :  :*:*

CcaD      QIARQRFDLGPFFTDTNIHSSMTVELTQGCSVGCWFCALSPDSFKENYNYDDNREEW-LG   167
PoyD      QIQRCEIELGSDQNTAIVHTPVVFELTRGCSVKCWFCALDAPPLTGIFDYSPENARFFRD   179
          ** * .::**   . : :*: :..***:**** ******.   :.  ::*. :. .:  .

CcaD      LLDTMHSFLGDAMKSTFLYWATEPFDNPDYEKFCLDVYESCGVFPPTTTAVAHKDTERIR   227
PoyD      VLRVVKDVIGPASKWASSYWGTDPLDNPDQEKFSLDFREILGMYPQTTTAIPLRAPERTR   239
          :* .::..:* * * :  **.*:*:**** ***.**. *  *::* ****:  :  ** *

CcaD      RFIRLSAEHGCWLNRFSLTSLGIMSKVHKAFSAEELAEVECLALNMNTSFAYGNAGNFRK   287
PoyD      KLLEVSHASGCLVNRFSVRTPAQLRKIHDTFSAEELLYTELVLQNLESDSVKARAGRLIH   299
          :::.:*   ** :****: : . : *:*.:******  .* :  *:::. . ..**.: :

CcaD      KALERPEILSQQDNKLRK------------------------APWHR--SNPD-YAGSE-   319
PoyD      FADDLPKLAAKDEEKLLNMLQEREPELAAKATSILINLPGSTEPIIRATSNADEEDTSEE   359
           * : *:: :::::** :                         *  *  ** *    **

CcaD      ----EYANGSICCVTGFLINAVSRKVQLISPTTASDEWPLGYIIFAEATYENAAGLEMIL   375
PoyD      YNVSINVPGTTSCLTGFKINMVDRTVELLSPCPANERWPLGHIVFEEGTFDTAEDLRTLM   419
                . *: .*:*** ** *.*.*:*:**  *.:.****:*:* *.*::.* .*. ::

CcaD      QSWSTEYFKEKLQDNDILRFHPWLGVSVSPDRIEIKGRFNQKEIIEESANPSLYHLIKQI   435
PoyD      LGMISRNMAERVVPESLVRFVPRLIYREDPEGFRLGSVFGNGVICHDPTRSAYLHRLGNL   479
           .  :. : *::  :.::** * *    .*: :.: . *.:  * .: :. :  * : ::

CcaD      -TEAPARLADIRQYAAAHLSIPPQVSDSLINELYKKGFFYGC--------------     476
PoyD      LREGKKRAGEIAMLCFYEFGVPENYTMGSINNMFHQGIIAEEPIATEAPIAVAAYQ     535
            *.  * .:*   .  .:.:* : : . **:::::*::
```

Supplementary Figure 6. Sequence alignment of CcaD and Radical SAM epimerase PoyD[4].

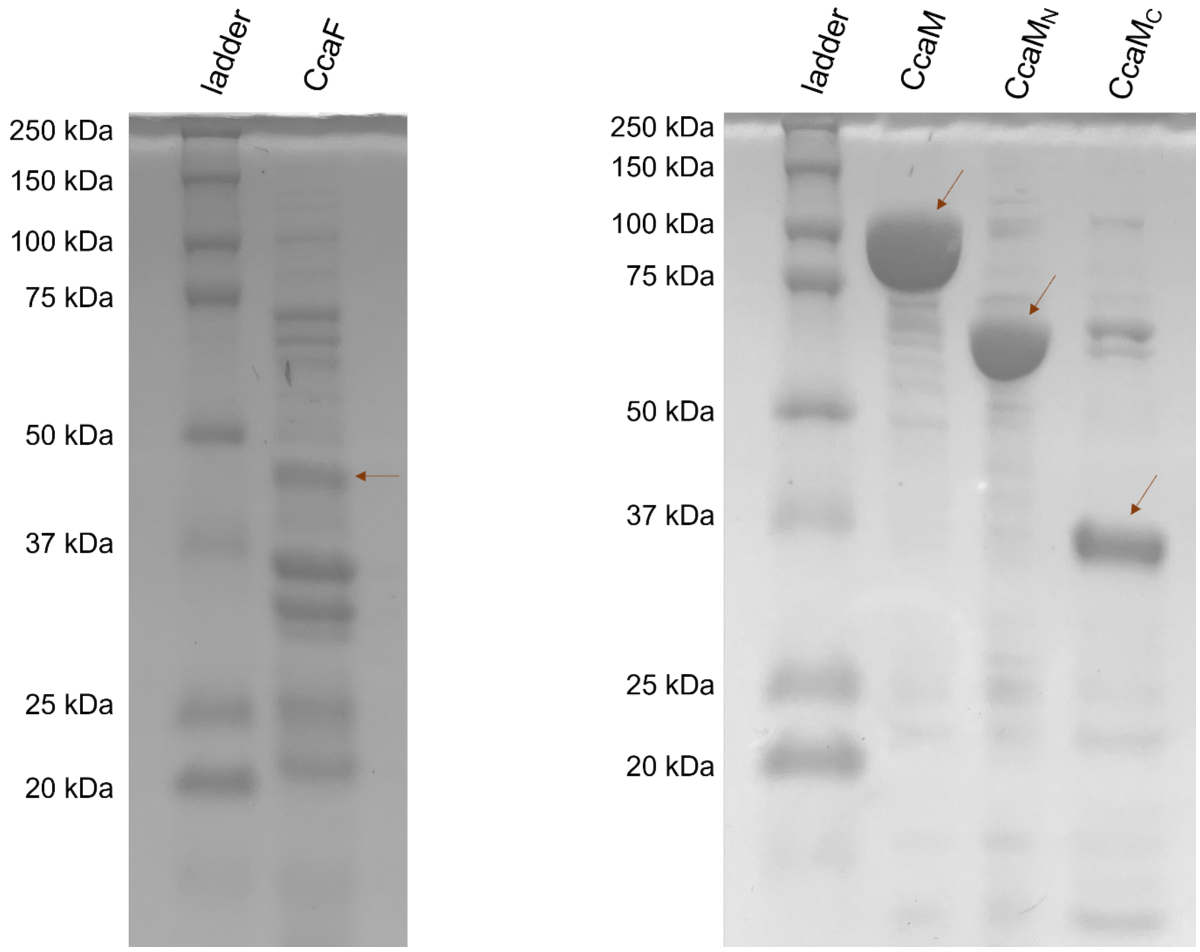

Supplementary Figure 7. SDS-PAGE analysis of purified proteins used in this study.

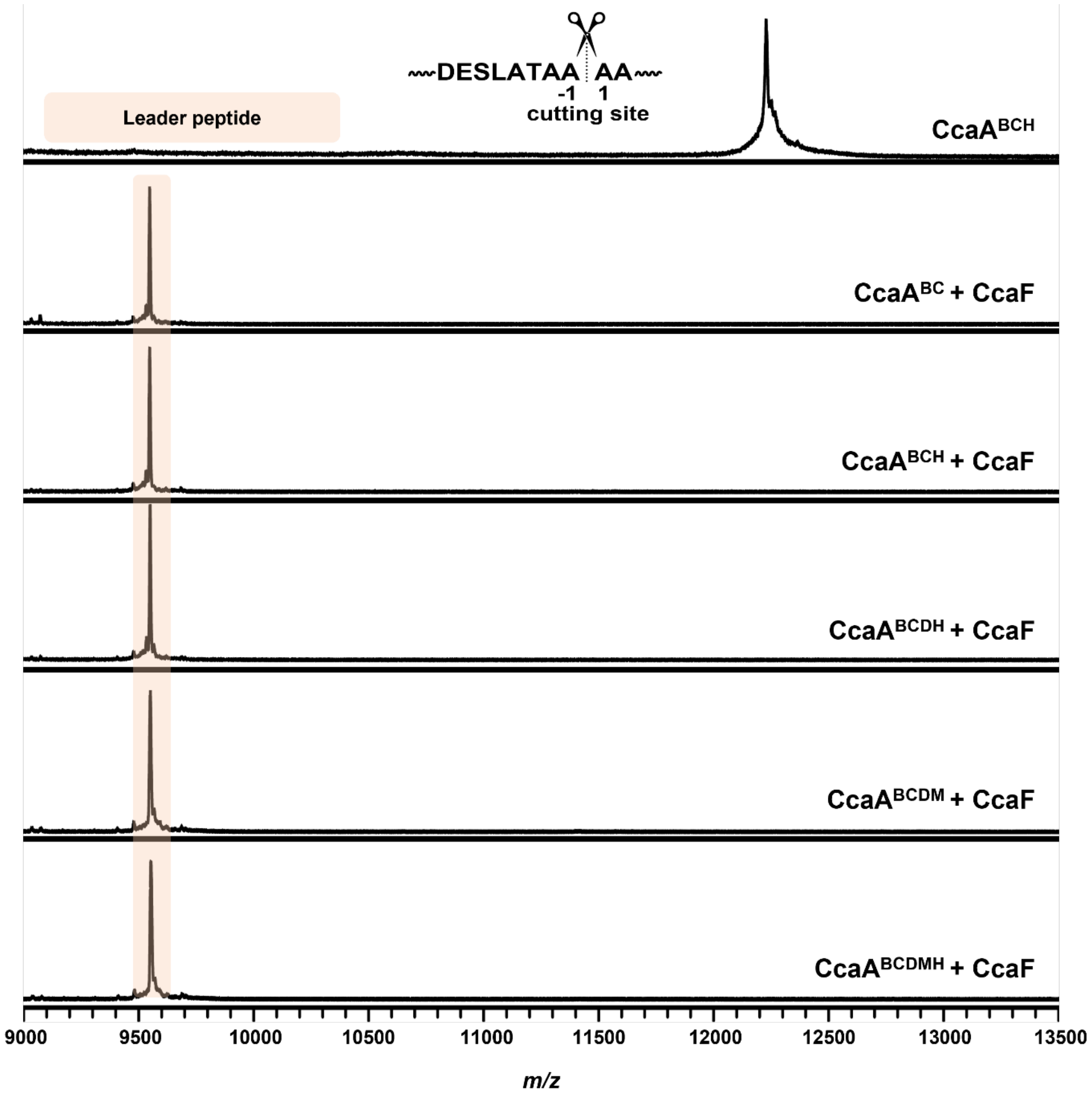

Supplementary Figure 8. In vitro removal of leader peptide of different modified precursor peptides by CcaF. The cutting site is shown in cartoon.

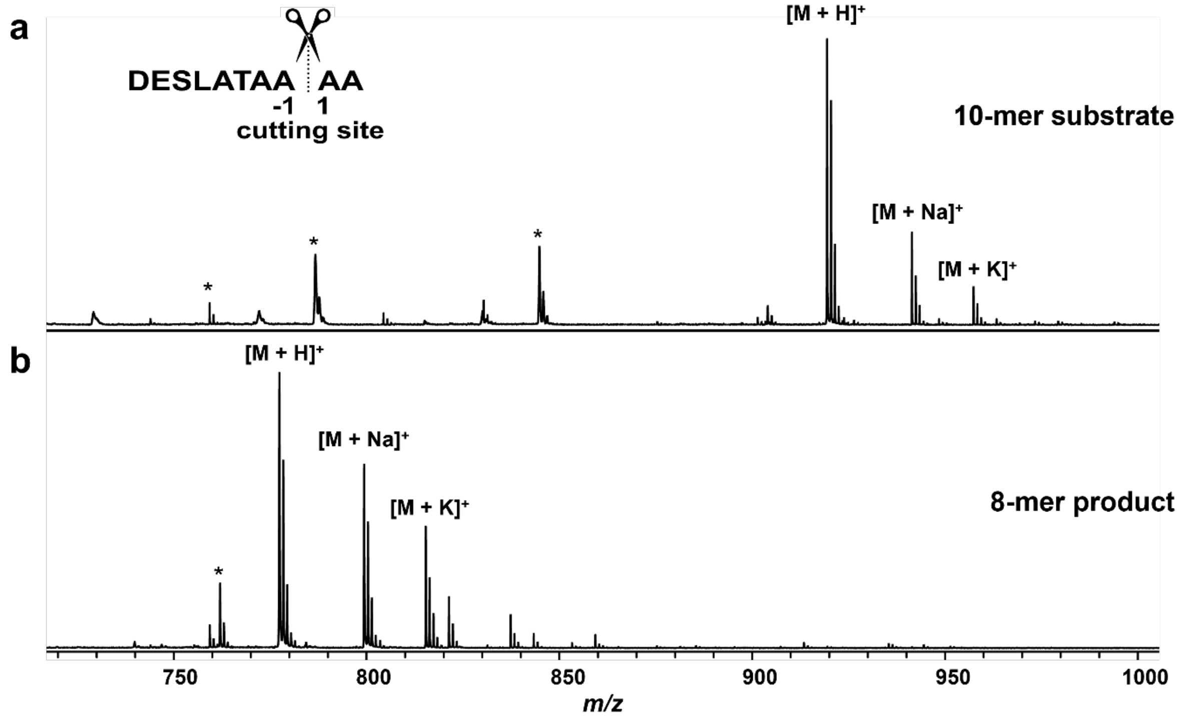

Supplementary Figure 9. **In vitro CcaF assay of a syntheic10-mer peptide (DELSATAAAA)**. (**a**) 10-mer peptide substrate. (**b**) In vitro CcaF assay of 10-mer peptide substrate. (∗) Denotes impurities which cannot be removed by HPLC.

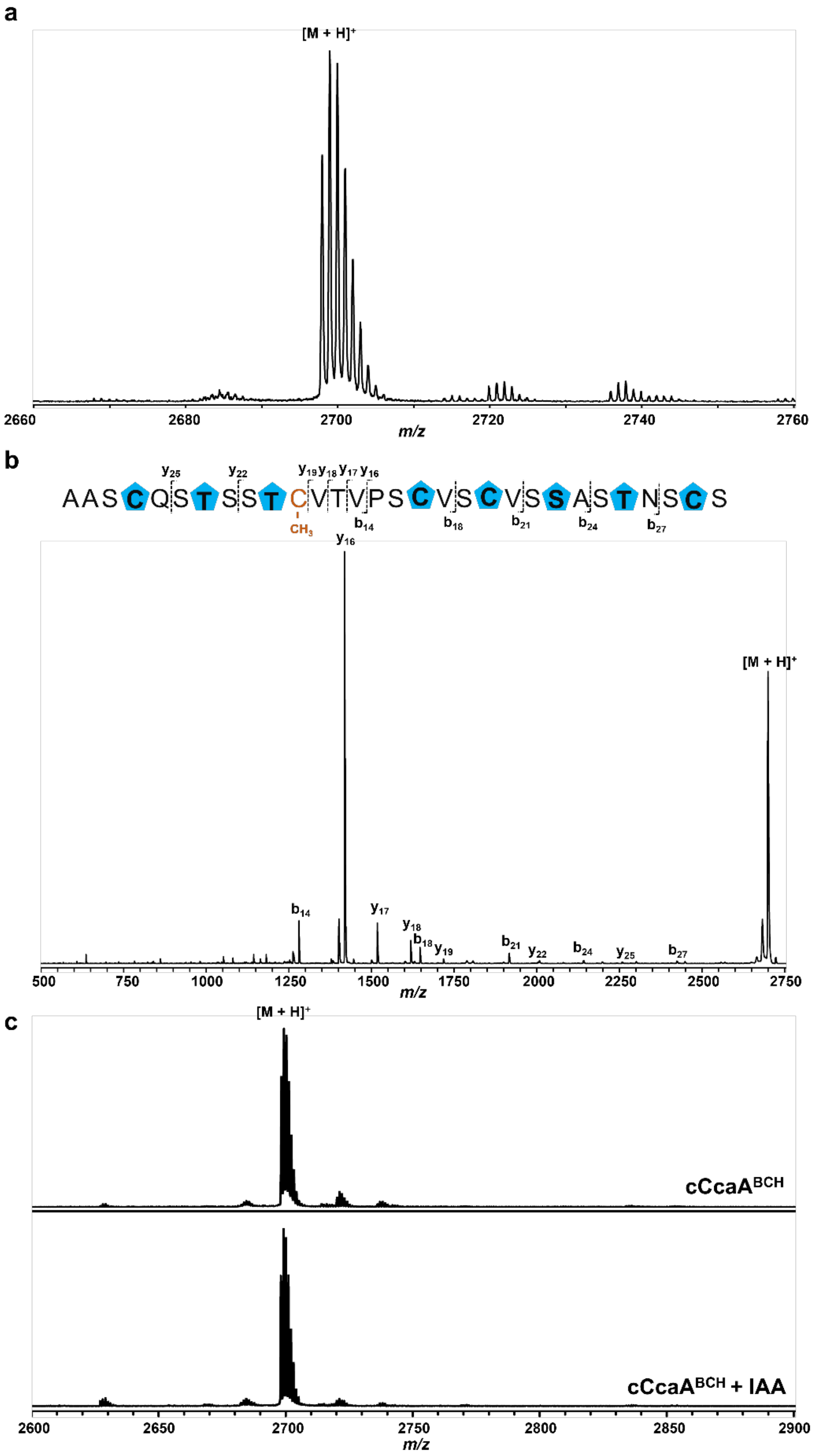

Supplementary Figure 10. **MALDI-TOF mass spectra of cCcaA^{BCH}**. (**a**) MALDI-TOF MS of cCcaA^{BCH}. (**b**) MALDI-TOF MS/MS of cCcaA^{BCH}. (**c**) IAA alkylation assay shows that no free cysteine in cCcaA^{BCH}.

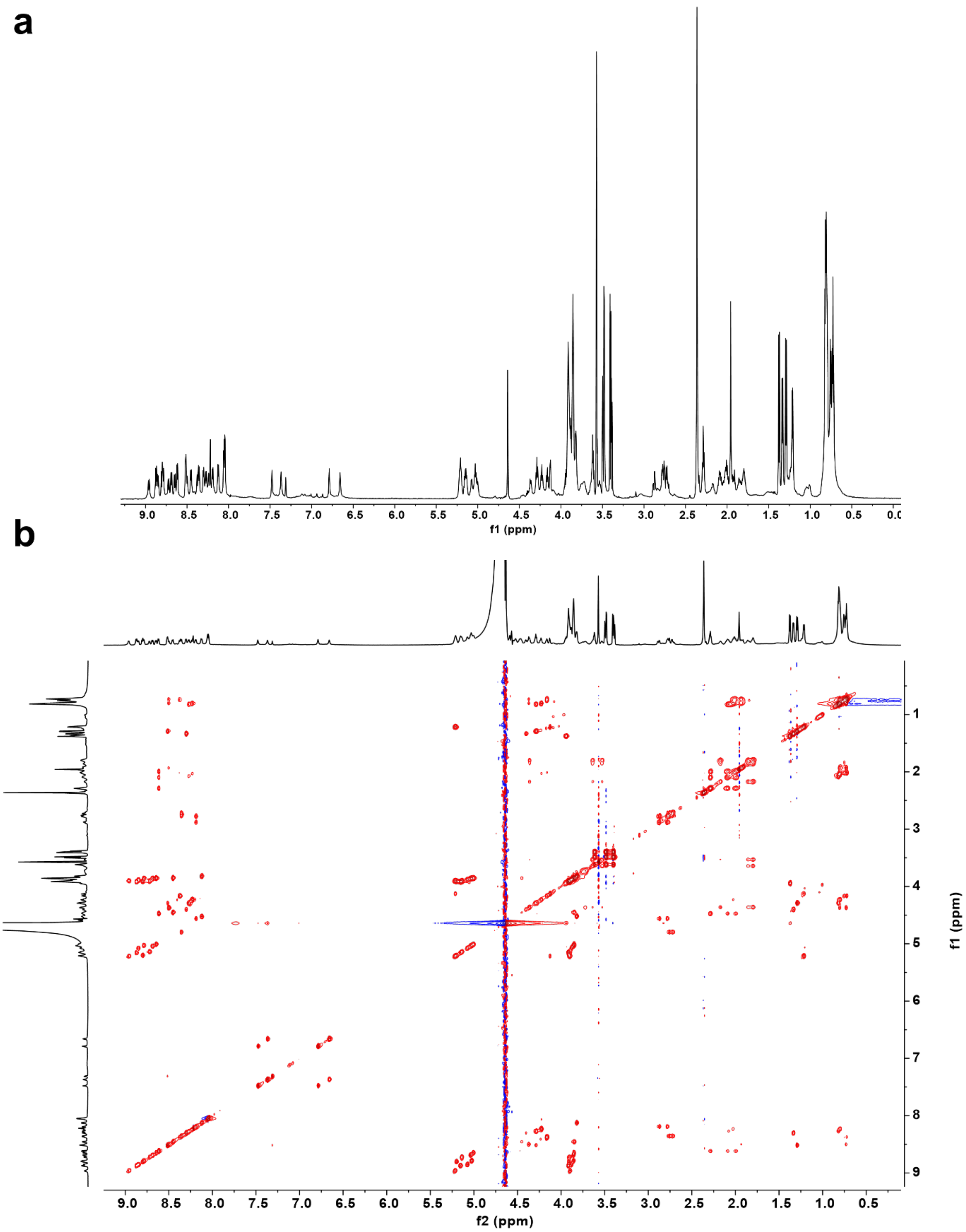

a
b
f1 (ppm)
f2 (ppm)
f1 (ppm)

c

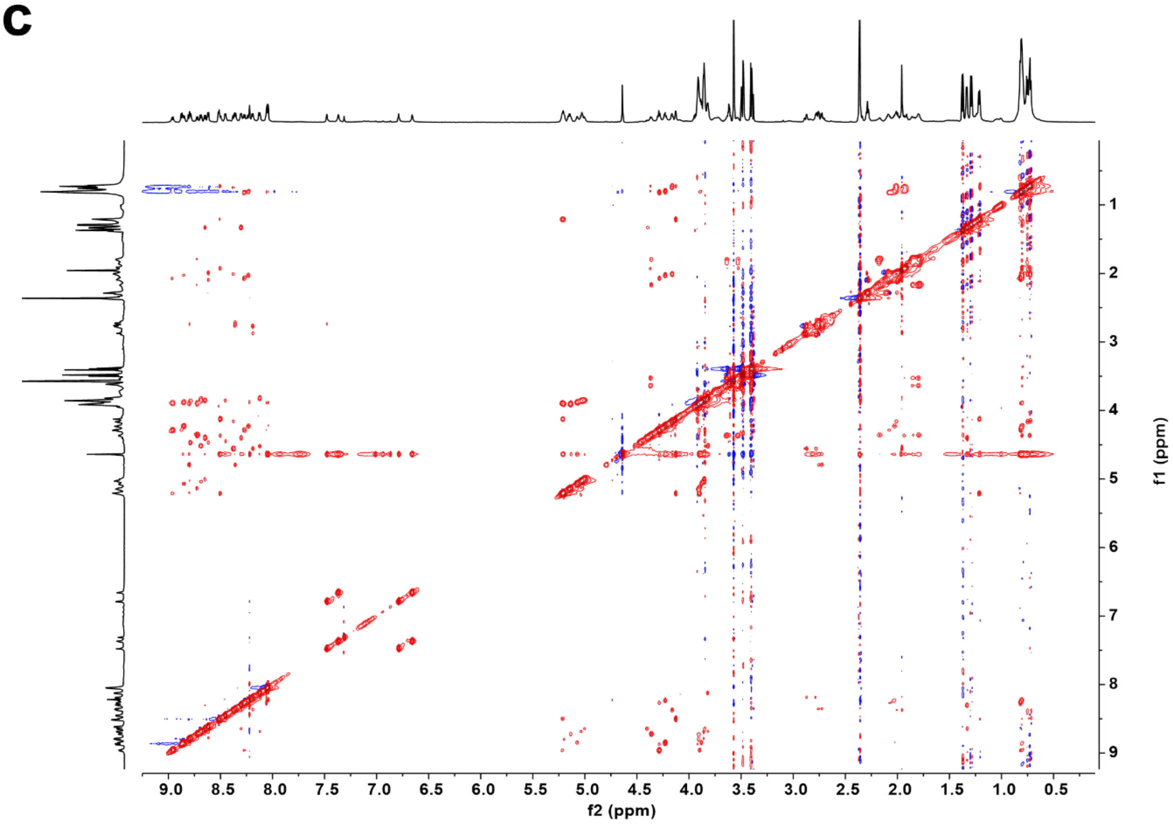
9.0 8.5 8.0 7.5 7.0 6.5 6.0 5.5 5.0 4.5 4.0 3.5 3.0 2.5 2.0 1.5 1.0 0.5
f2 (ppm)
1 2 3 4 5 6 7 8 9
f1 (ppm)

d

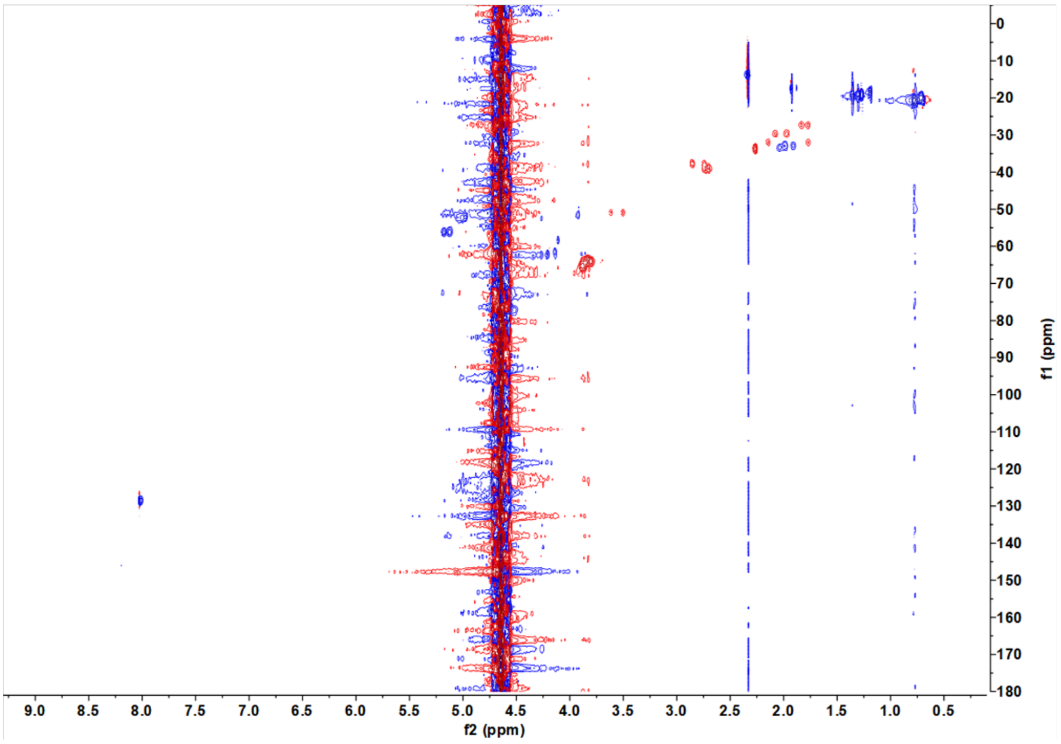
9.0 8.5 8.0 7.5 7.0 6.5 6.0 5.5 5.0 4.5 4.0 3.5 3.0 2.5 2.0 1.5 1.0 0.5
f2 (ppm)
0 10 20 30 40 50 60 70 80 90 100 110 120 130 140 150 160 170 180
f1 (ppm)

e

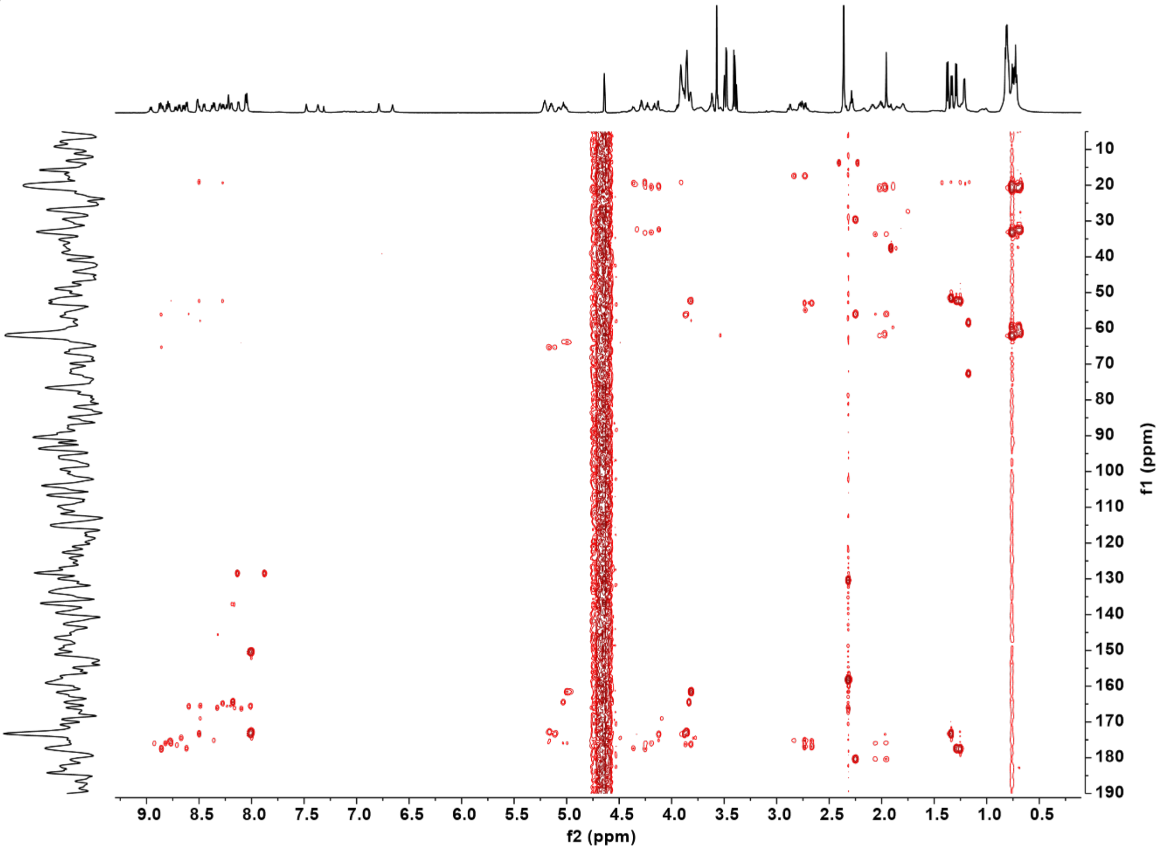

Supplementary Figure 11. **NMR spectra of cCcaA^{BCH} in 9:1 (v/v) H_2O/D_2O at 25 °C**. (**a**) ^{1}H NMR spectrum. (**b**) ^{1}H-^{1}H TOCSY spectrum. (**c**) ^{1}H-^{1}H NOESY spectrum. (**d**) ^{1}H-^{13}C HSQC spectrum. (**e**) ^{1}H-^{13}C HMBC spectrum.

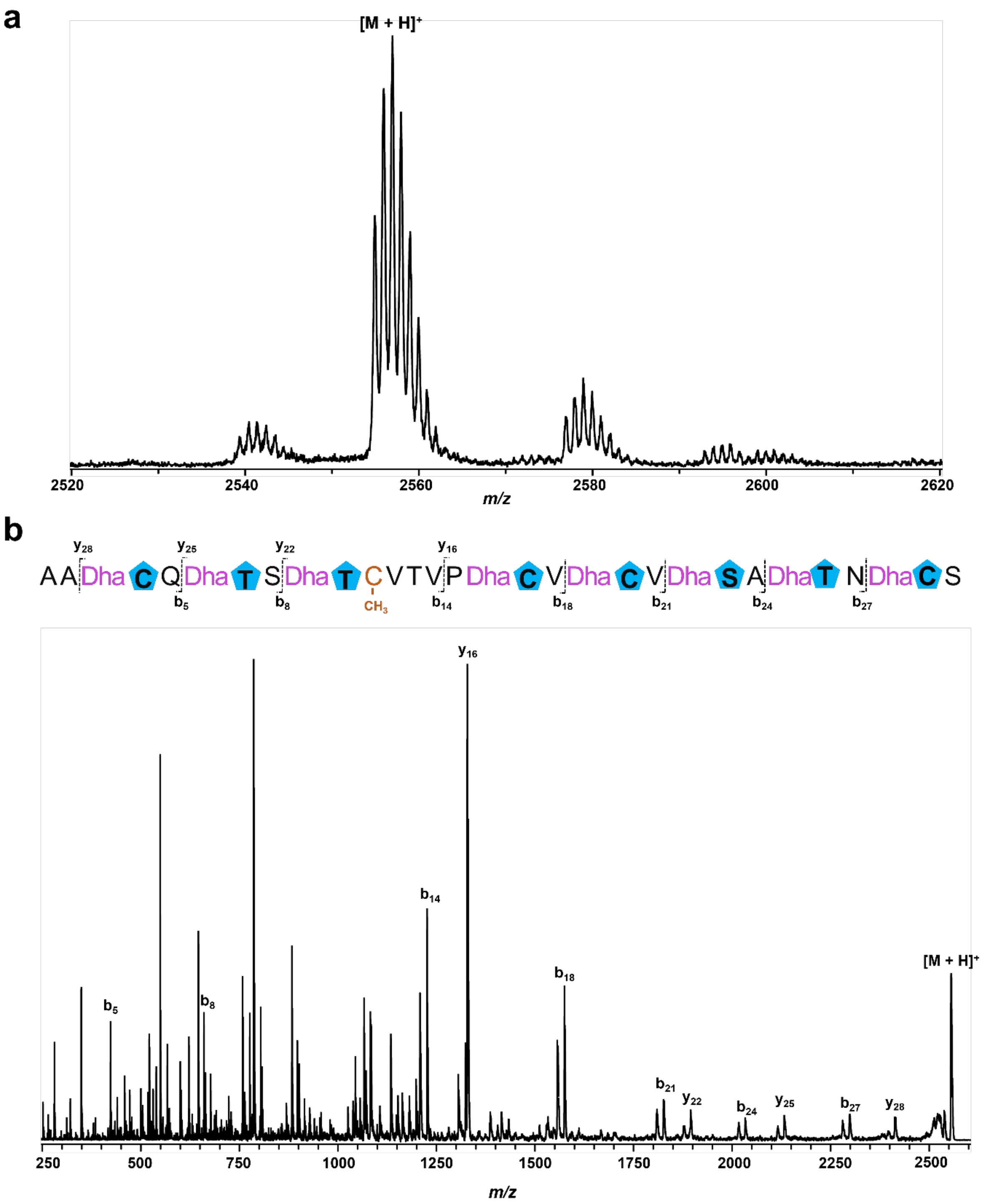

Supplementary Figure 12. **MALDI-TOF mass spectra of carnazolamide**. (**a**) MALDI-TOF MS of carnazolamide. (**b**) MALDI-TOF MS/MS of carnazolamide.

a

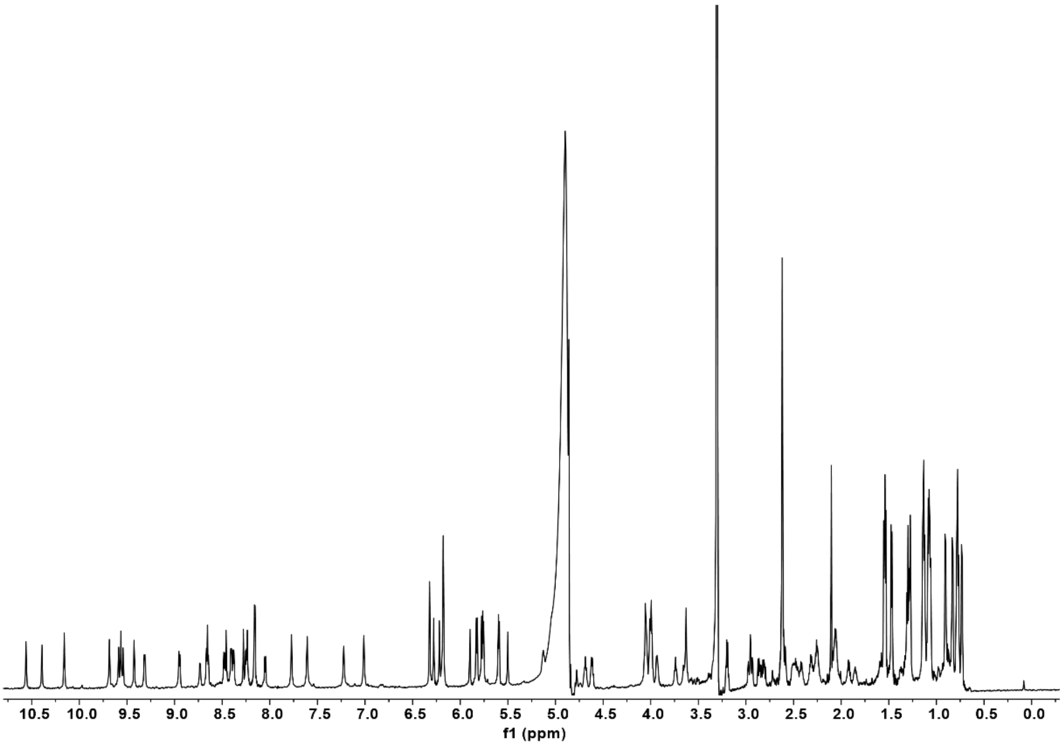
10.5
10.0
9.5
9.0
8.5
8.0
7.5
7.0
6.5
6.0
5.5
5.0
4.5
4.0
3.5
3.0
2.5
2.0
1.5
1.0
0.5
0.0
f1 (ppm)

b

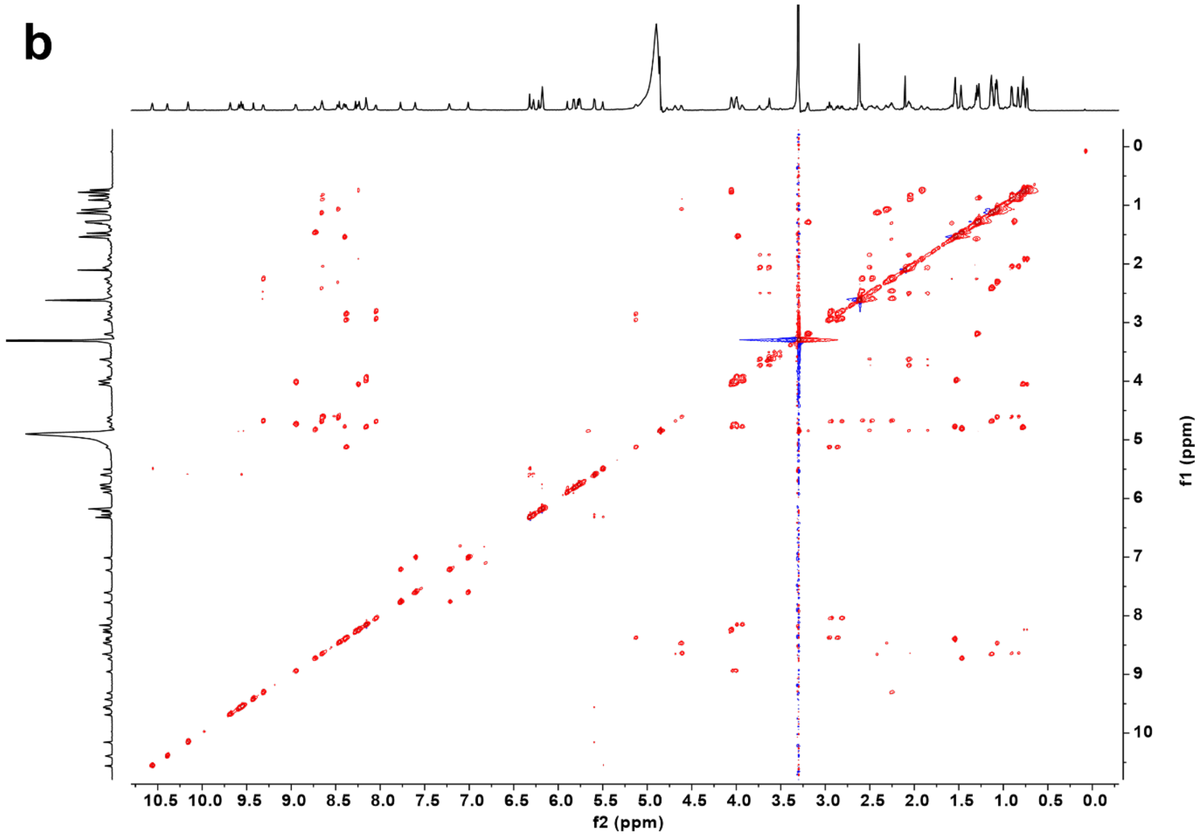
f2 (ppm)
f1 (ppm)

c

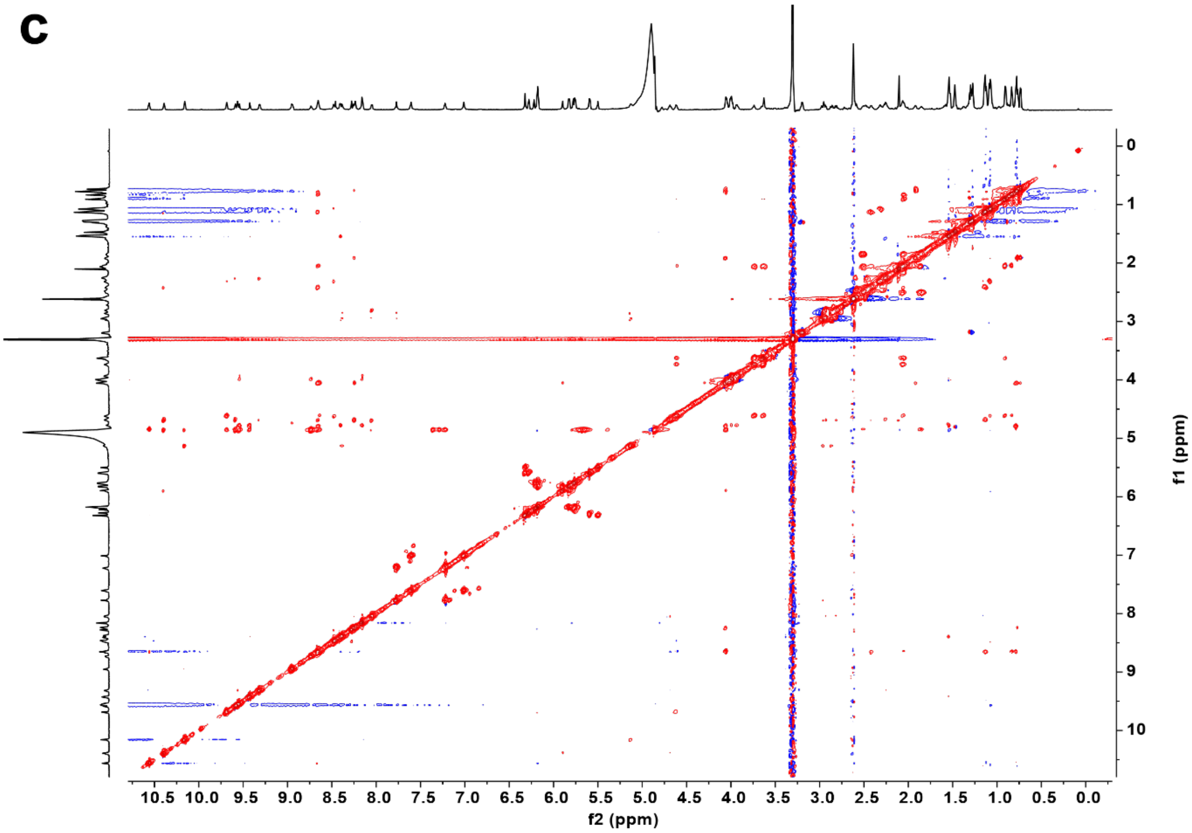

Supplementary Figure 13. NMR spectra of carnazolamide in CD_3OH at 25 °C. (**a**) ^1H NMR spectrum. (**b**) ^1H-^1H TOCSY spectrum. (**c**) ^1H-^1H NOESY spectrum.

a

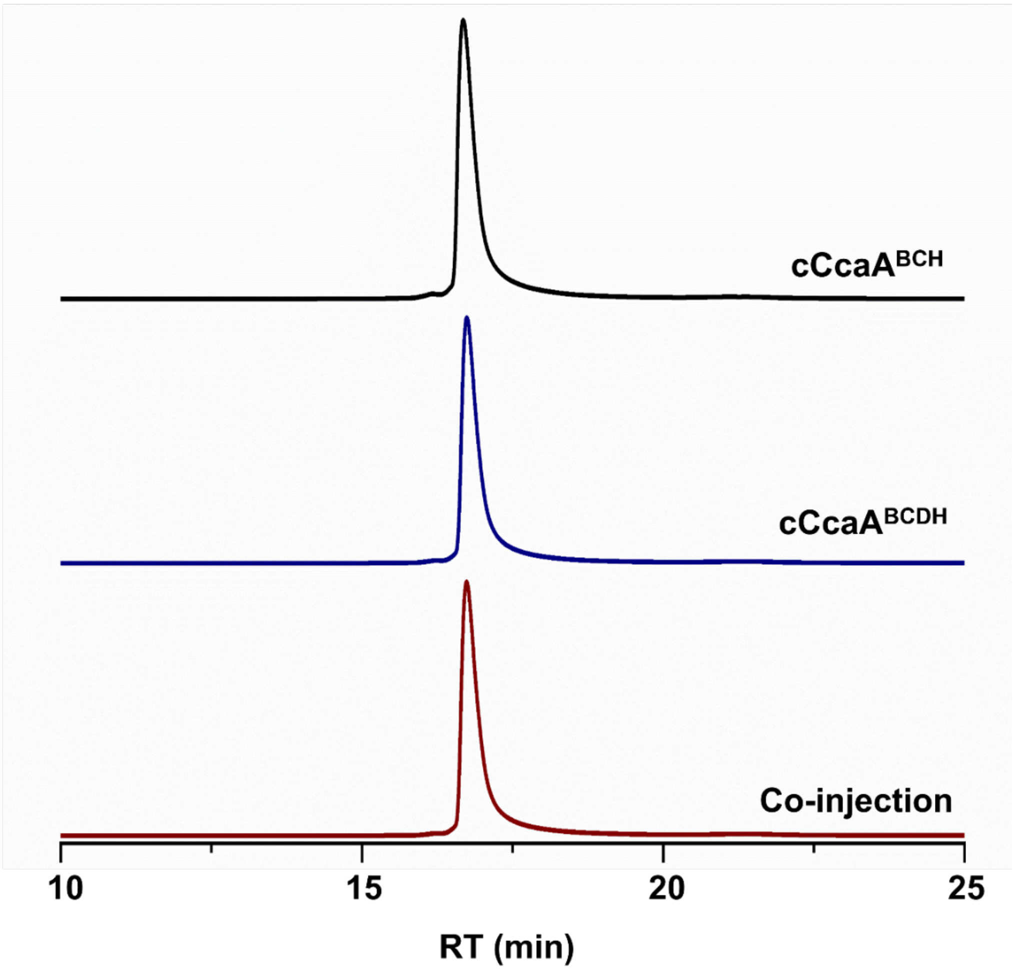

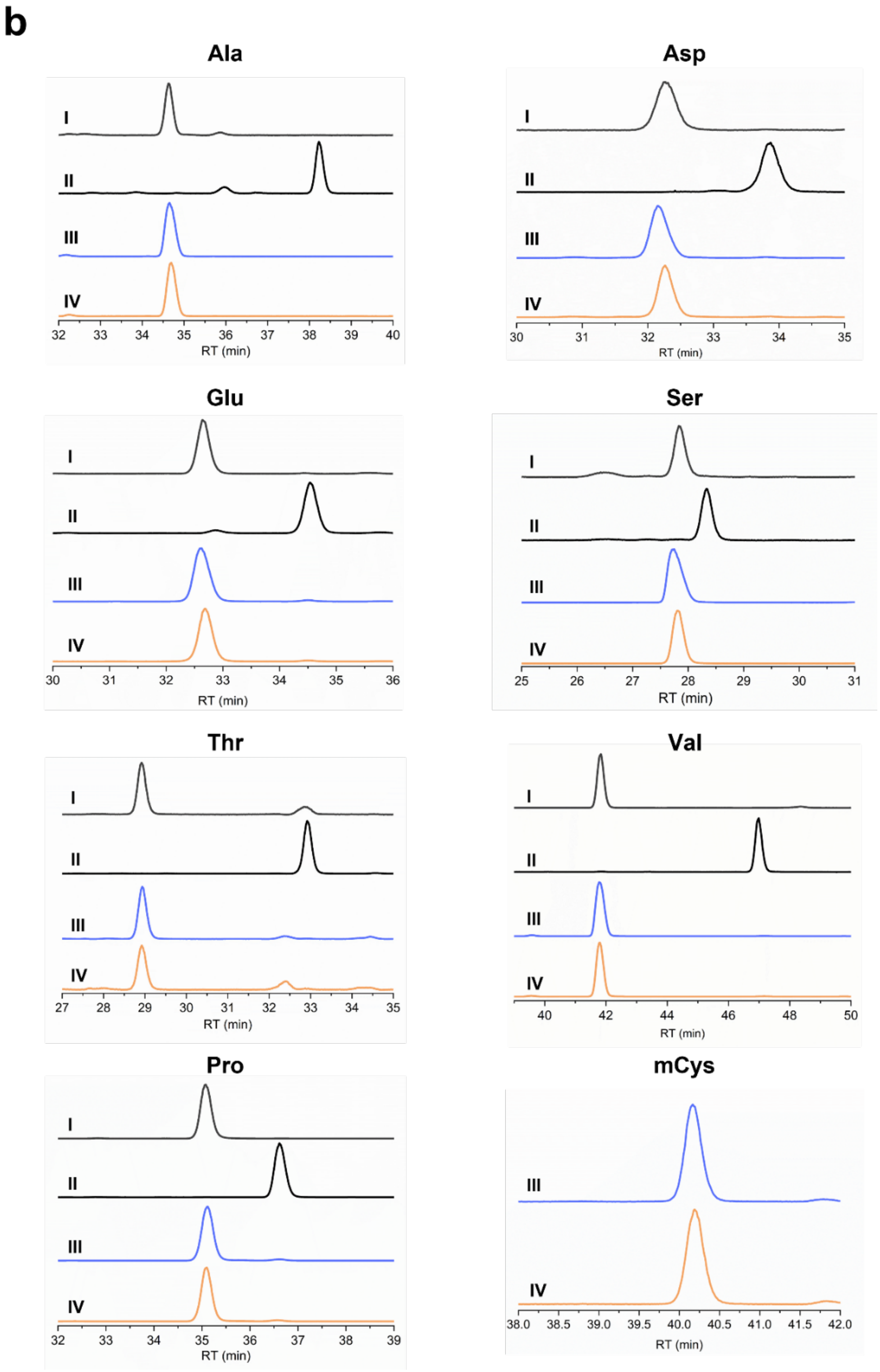

Supplementary Figure 14. HPLC analysis cCcaA^{BCH} and cCcaA^{BCDH} and Marfey's assay of cCcaA^{BCH} hydrolysate and cCcaA^{BCDH} hydrolysate. (**a**) HPLC analysis of cCcaA^{BCH} and cCcaA^{BCDH}. (**b**) Marfey's assay of cCcaA^{BCH} hydrolysate and cCcaA^{BCDH} hydrolysate. L-amino acid standard (I), D-amino acid standard (II), cCcaA^{BCH} hydrolysate (III) and cCcaA^{BCDH} hydrolysate (IV). mCys means *S*-methylated cysteine.

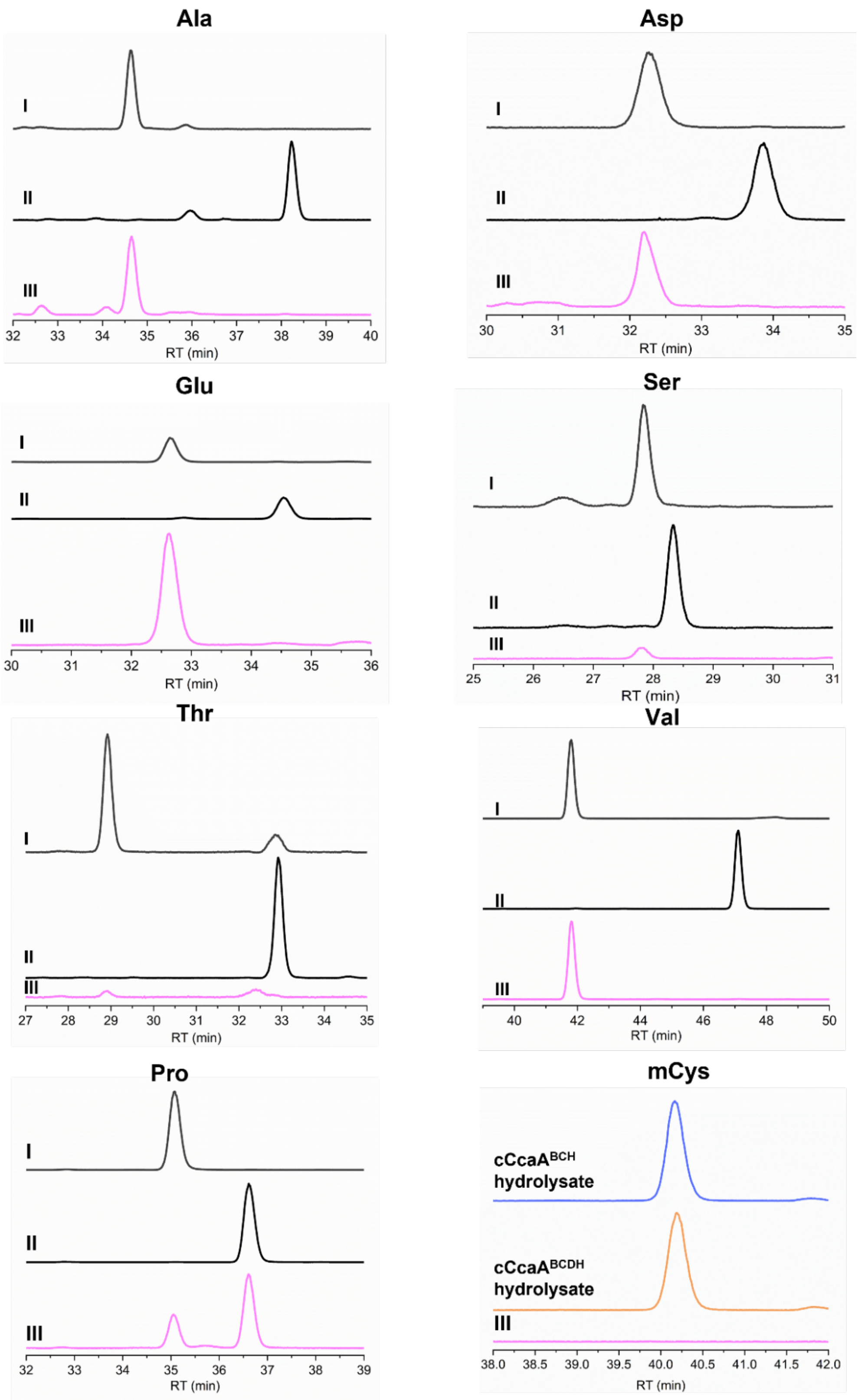

Supplementary Figure 15. Marfey's assay of carnazolamide hydrolysate. L-amino acid standard (I), D-amino acid standard (II) and carnazolamide hydrolysate (III). The *S*-methylated cysteine was not shown for the intensity is too low to be detected.

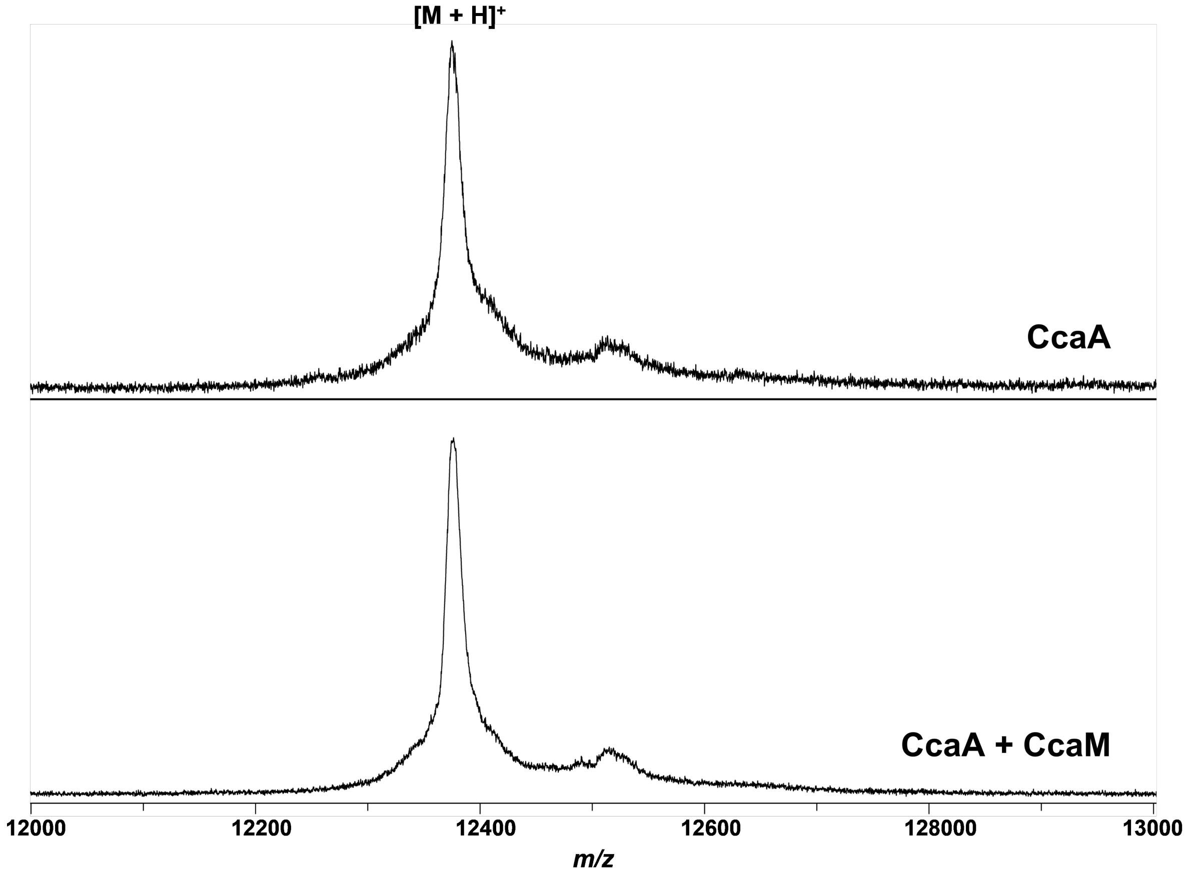

Supplementary Figure 16. In vitro assay of full length CcaA with CcaM. CcaM does not catalyze dehydration of the unmodified precursor peptide.

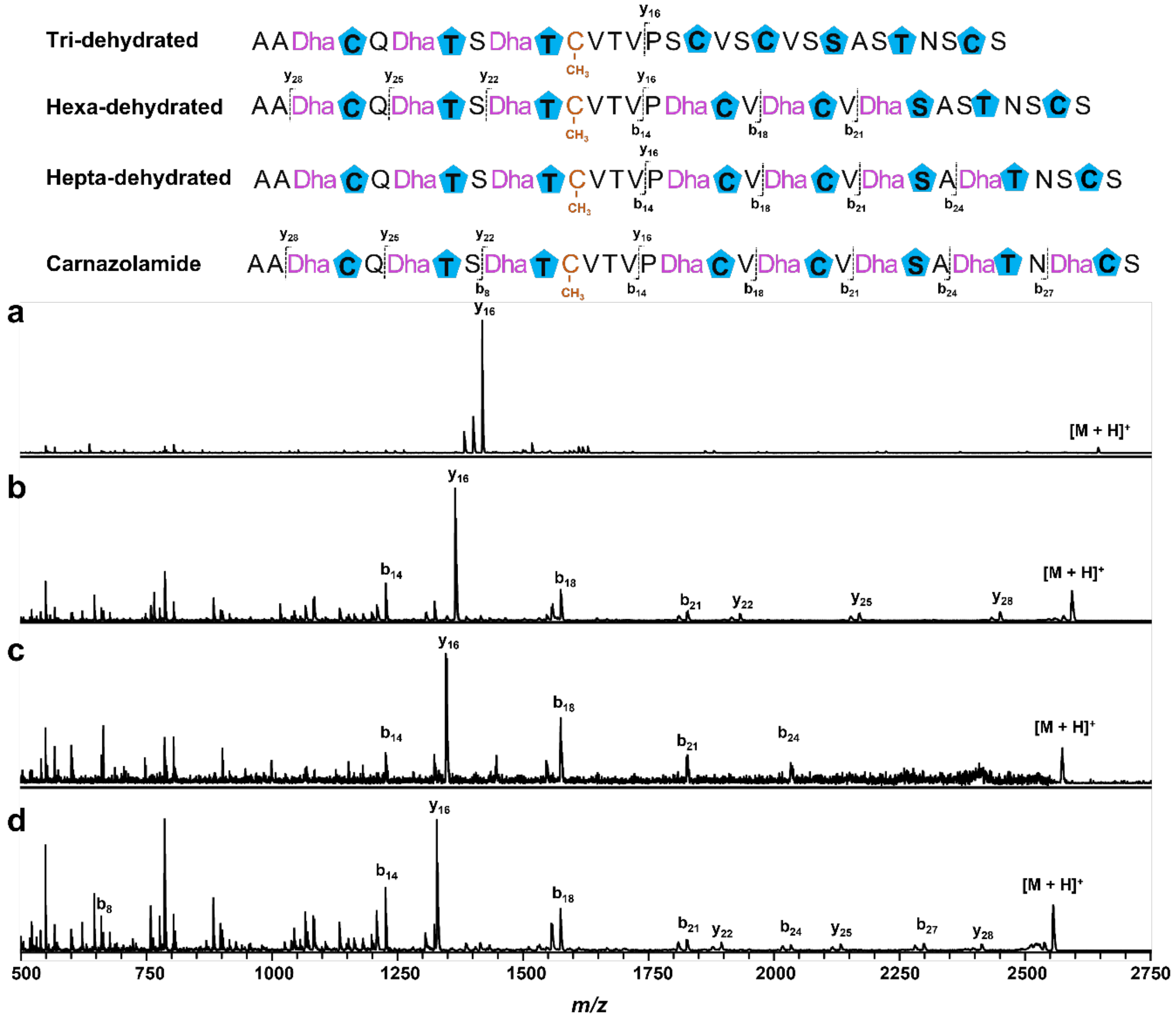

Supplementary Figure 17. MALDI-TOF MS/MS of the tri (**a**), hexa (**b**), hepta (**c**) dehydrated ocat-azole containing core peptide and carnazolamide (**d**).

a

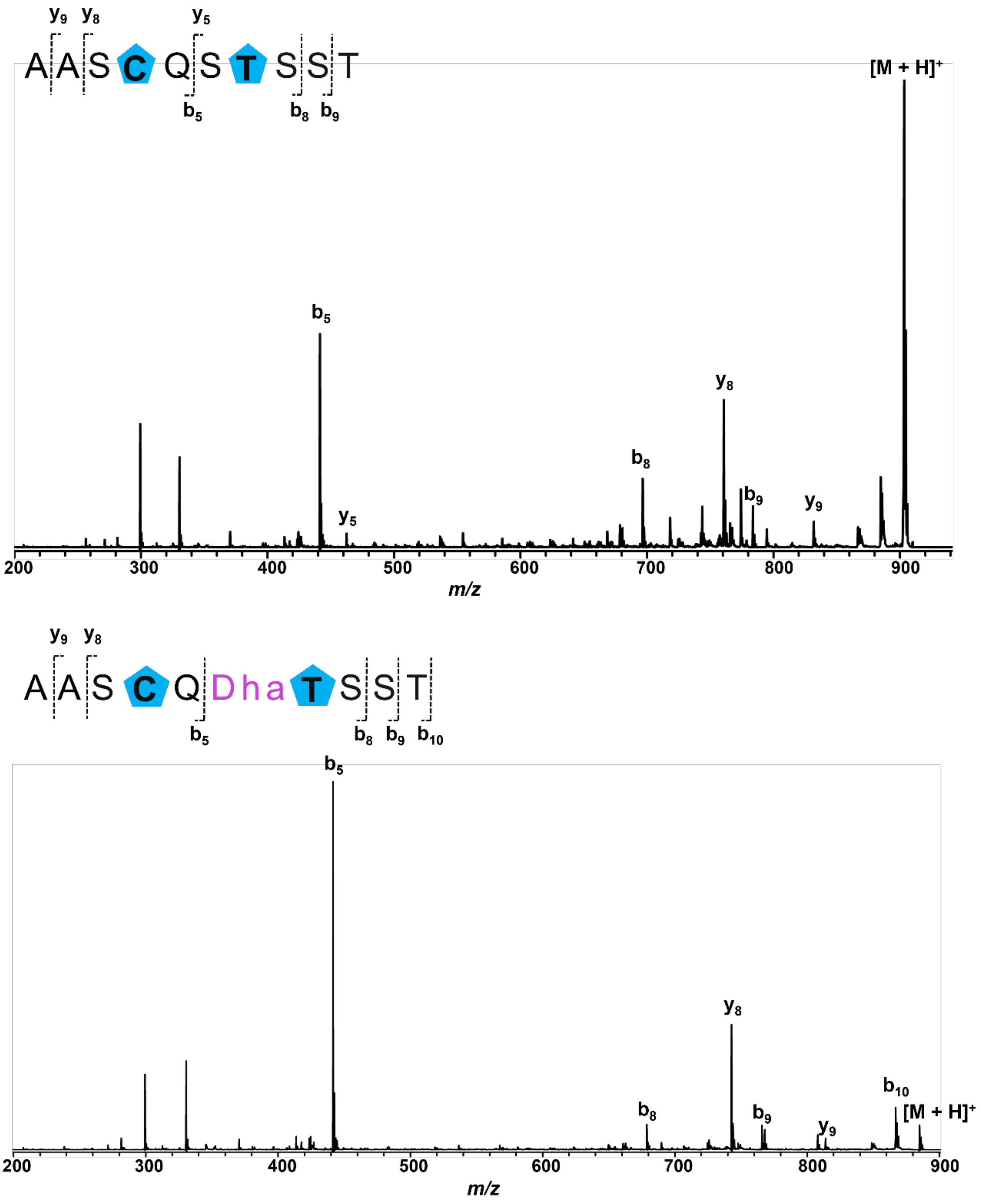

y9 y8 y5
AASCQSTSST
b5 b8 b9
[M + H]+
b5
y8
b8
b9
y9
y5
200 300 400 500 600 700 800 900
m/z
y9 y8
AASCQDhaTSST
b5 b8 b9 b10
b5
y8
b10
[M + H]+
b8
b9
y9
200 300 400 500 600 700 800 900
m/z

b

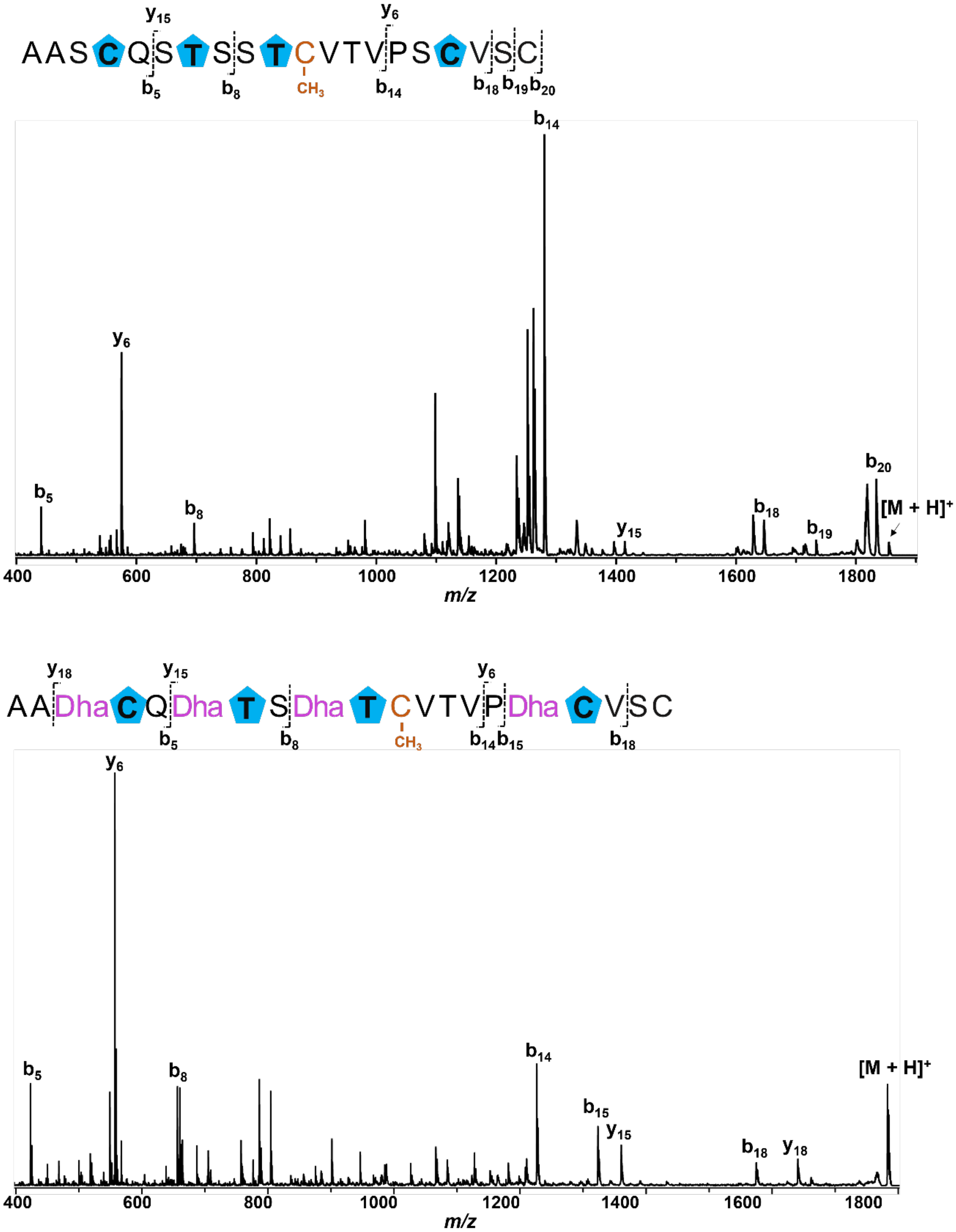

AASCQSTSSTCVTVPSCVSC
CH3
y15 y6
b5 b8 b14 b18 b19 b20
b5 y6 b8 b14 y15 b18 b19 b20 [M + H]+
400 600 800 1000 1200 1400 1600 1800
m/z
AADhaCQDhaTSDhaTCVTVPDhaCVSC
CH3
y18 y15 y6
b5 b8 b14 b15 b18
y6 b5 b8 b14 b15 y15 b18 y18 [M + H]+
400 600 800 1000 1200 1400 1600 1800
m/z

c

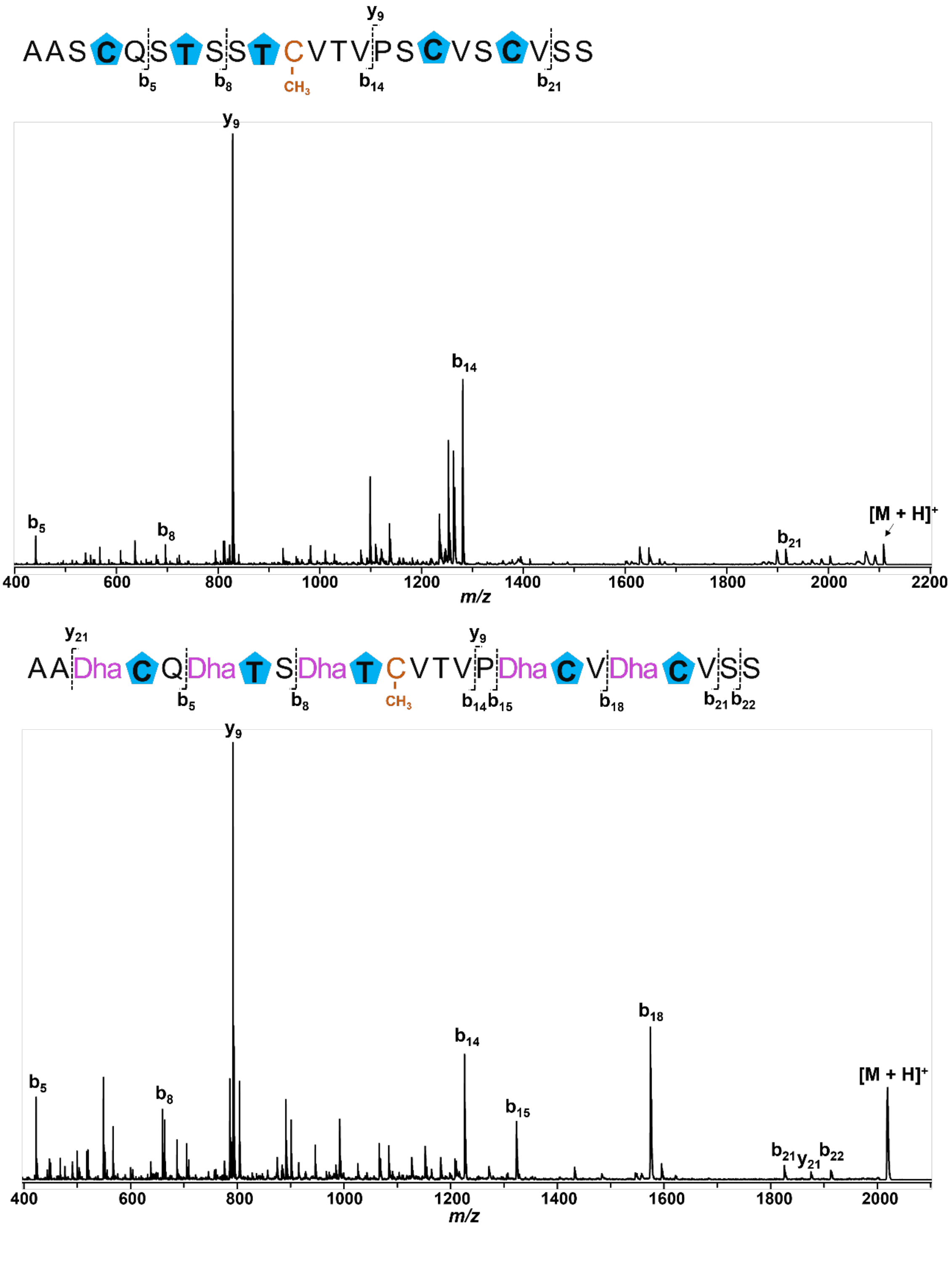

AASCQSTSSTCVTVPSCVSCVSS
CH3
y9
b5
b8
b14
b21
[M + H]+
m/z
400
600
800
1000
1200
1400
1600
1800
2000
2200
AADhaCQDhaTSDhaTCVTVPDhaCVDhaCVSS
CH3
y21
y9
b5
b8
b14
b15
b18
b21
b22
[M + H]+
m/z

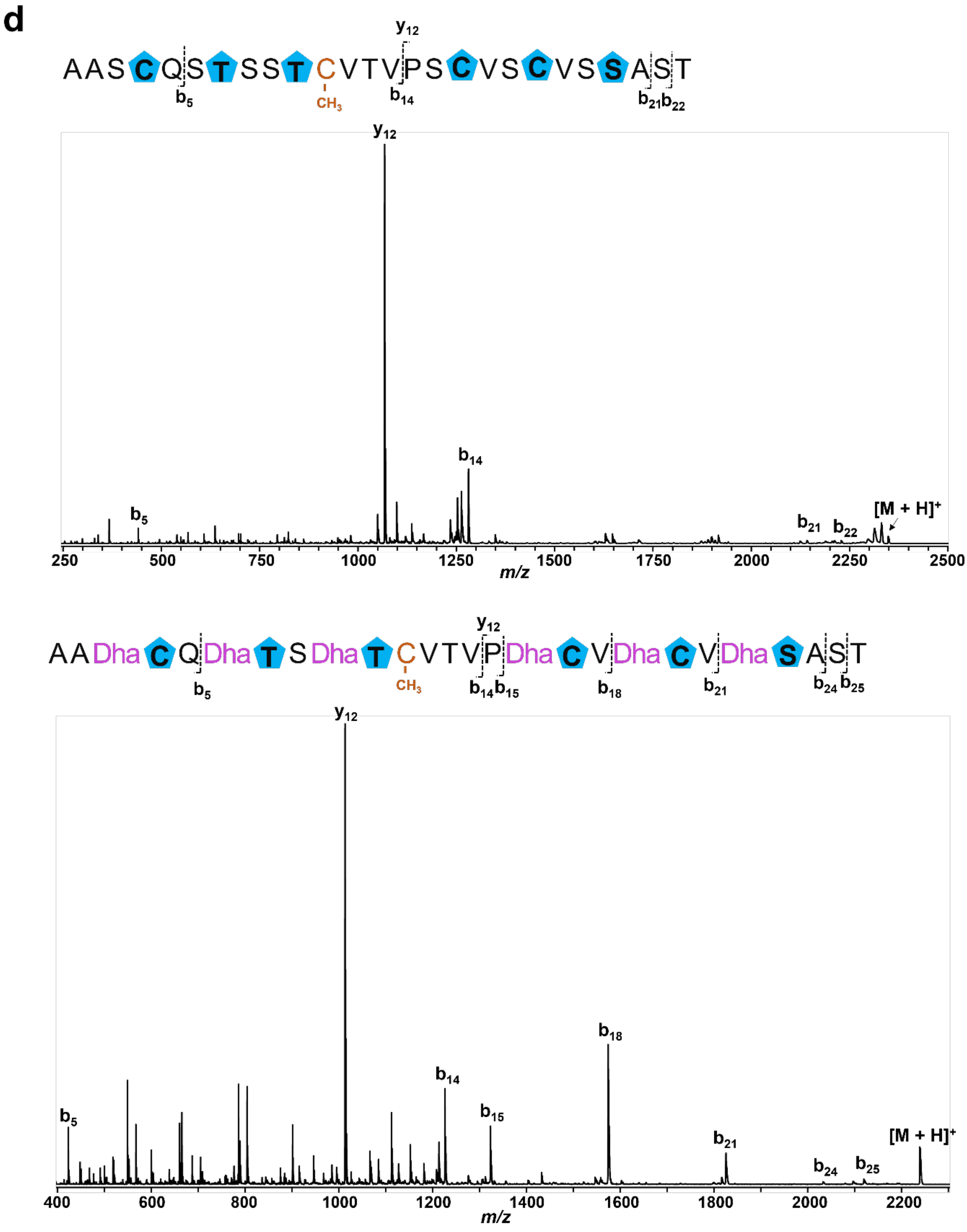

Supplementary Figure 18. MALDI-TOF MS/MS of cCcaA^{BCH} truncants and in vitro CcaM catalyzed dehydrated products. (**a**) MALDI-TOF MS/MS spectrum of cCcaA(1-10)^{BCH} and mono-dehydrated cCcaA(1-10)^{BCH}. (**b**) MALDI-TOF MS/MS spectrum of cCcaA(1-20)^{BCH} and tetra-dehydrated cCcaA(1-20)^{BCH}. (**c**) MALDI-TOF MS/MS spectrum of cCcaA(1-23)^{BCH} and penta-dehydrated cCcaA(1-23)^{BCH}. (**d**) MALDI-TOF MS/MS spectrum of cCcaA(1-26)^{BCH} and hexa-dehydrated cCcaA(1-26)^{BCH}.

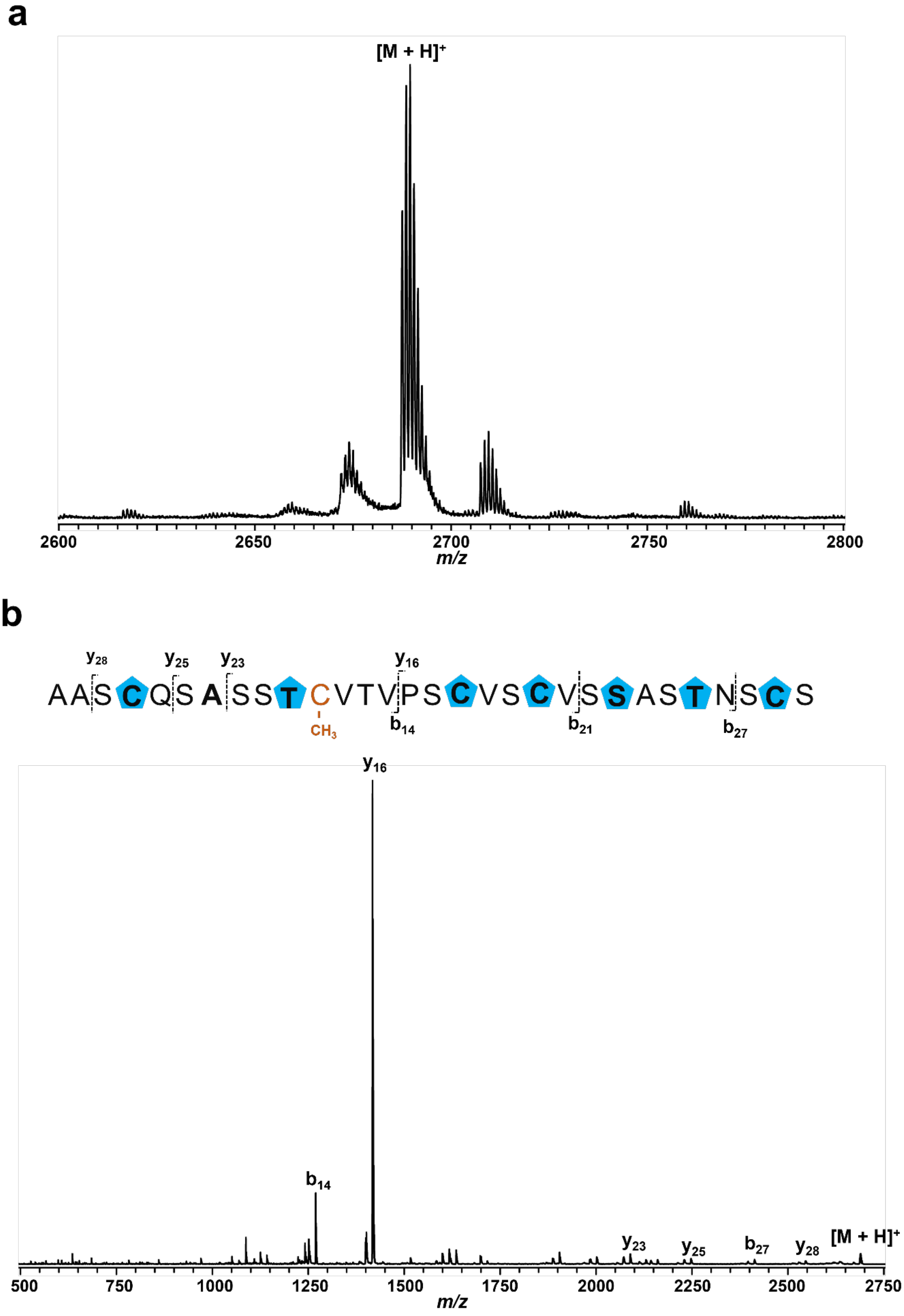

Supplementary Figure 19. MALDI-TOF mass spectra of cCcaA(T7A)^{BCH}. (**a**) MALDI-TOF MS of cCcaA(T7A)^{BCH}. **(b)** MALDI-TOF MS/MS of cCcaA(T7A)^{BCH}.

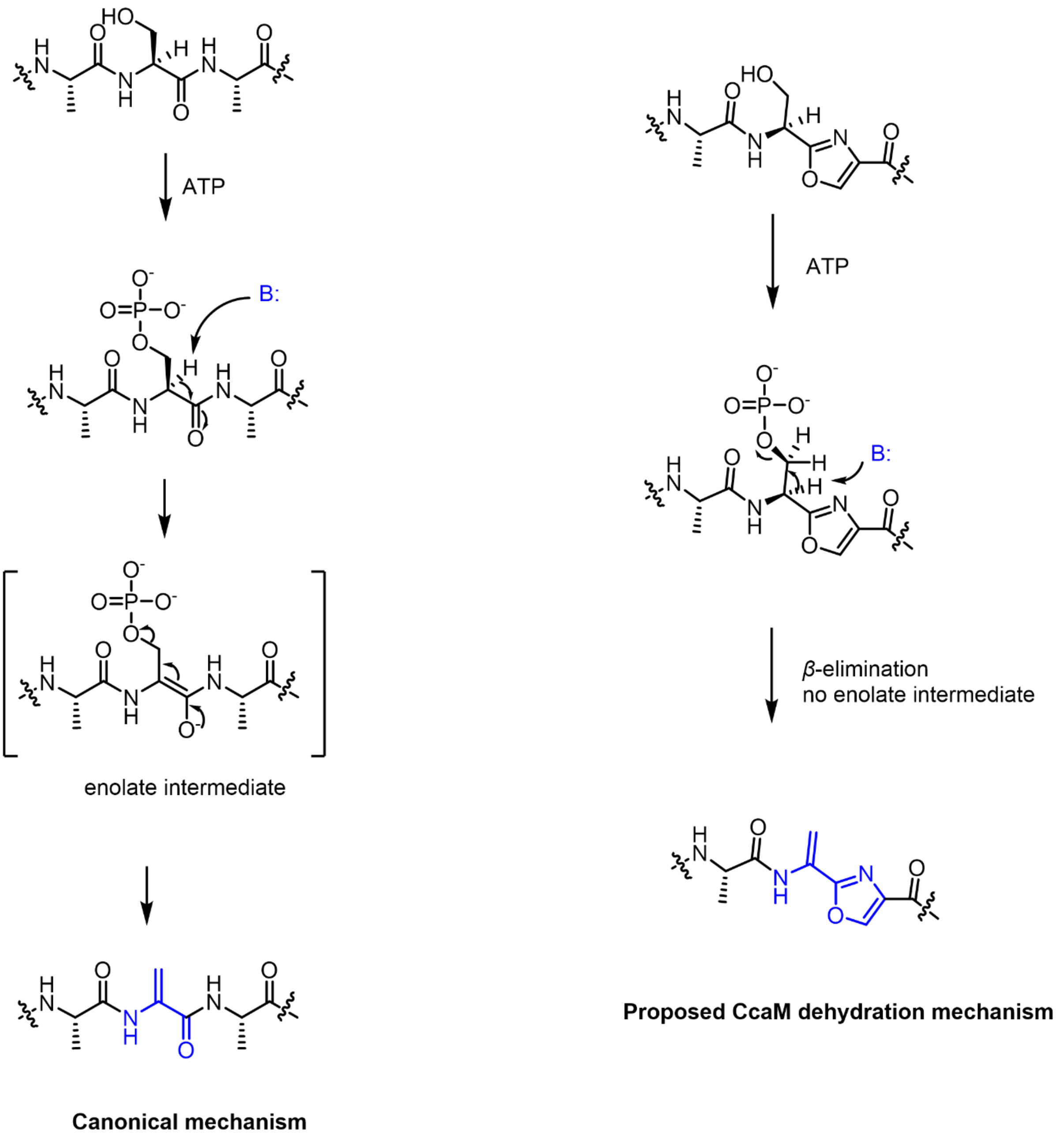

Supplementary Figure 20. Proposed dehydration mechanism of CcaM.

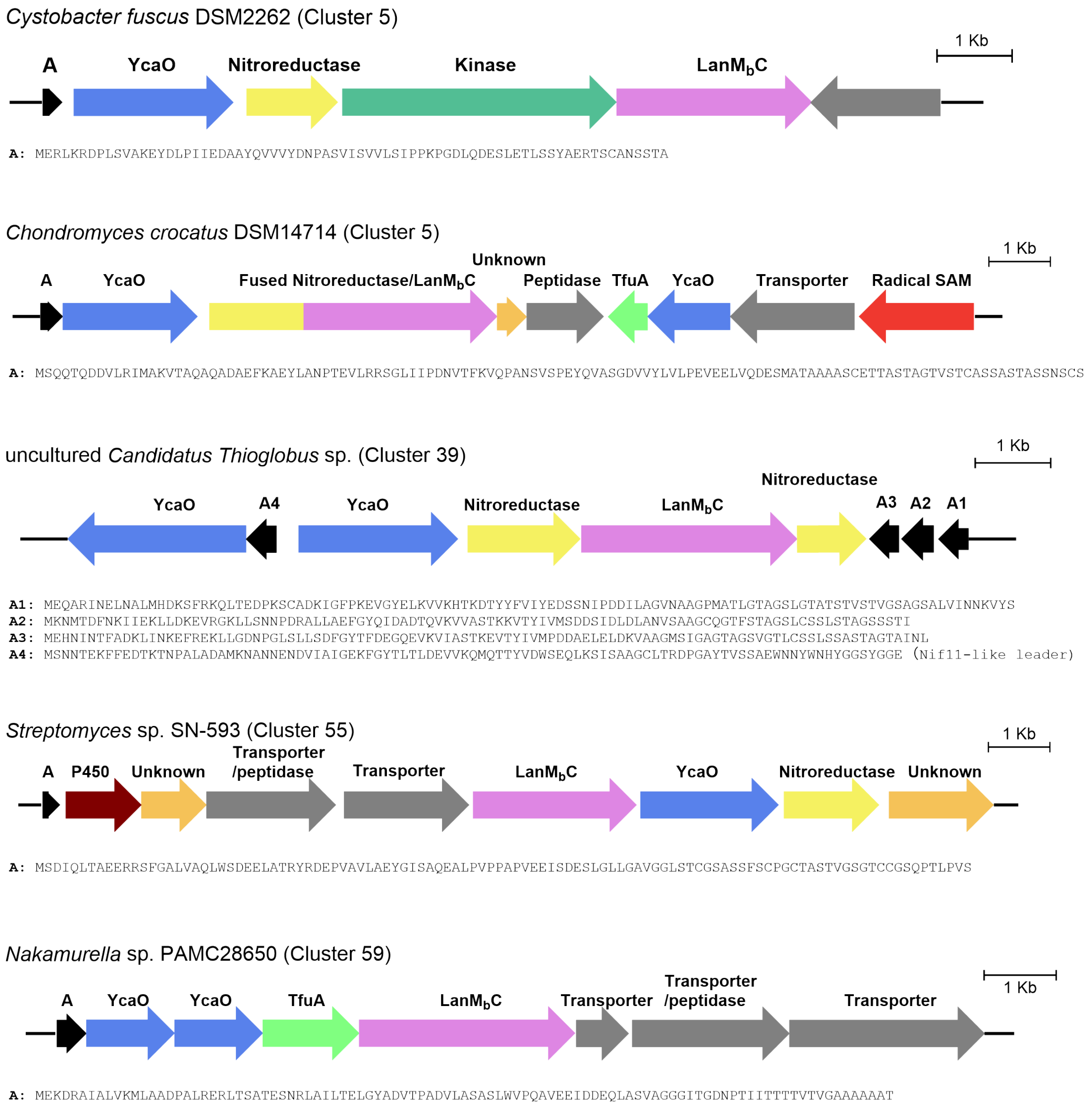

Supplementary Figure 21. Representative biosynthetic gene cluster containing the novel LanM$_b$C and a YcaO.

Supplementary Table 1. Plasmids constructed in this study.

Plasmid	Insert gene	Vector	Cloning site
pCcaA-RSF	*ccaA*	pRSF-Duet-TEV	*Bam*HI
pCcaAH-RSF	*ccaA*, *ccaH*	pRSF-Duet-TEV	*Bam*HI, *Nde*I
pCcaAD-RSF	*ccaA*, *ccaD*	pRSF-Duet-TEV	*Bam*HI, *Nde*I
pCcaAM-RSF	*ccaA*, *ccaM*	pRSF-Duet-TEV	*Bam*HI, *Nde*I
pCcaA(1-10)H-RSF	*ccaA (*1-10*)*, *ccaH*	pRSF-Duet-TEV	*Bam*HI, *Nde*I
pCcaA(1-20)H-RSF	*ccaA (1-20)*, *ccaH*	pRSF-Duet-TEV	*Bam*HI, *Nde*I
pCcaA(1-23)H-RSF	*ccaA (1-23)*, *ccaH*	pRSF-Duet-TEV	*Bam*HI, *Nde*I
pCcaA(1-26)H-RSF	*ccaA (1-26)*, *ccaH*	pRSF-Duet-TEV	*Bam*HI, *Nde*I
pCcaA(T7A)H-RSF	*ccaA (T7A)*, *ccaH*	pRSF-Duet-TEV	*Bam*HI, *Nde*I
pCcaA(M1C)H-RSF	*ccaA (M1C)*, *ccaH*	pRSF-Duet-TEV	*Bam*HI, *Nde*I
pCcaBC-CDF	*ccaB*, *ccaC*	pCDF-Duet-TEV	*Nco*I, *Nde*I
pCcaM-ET	*ccaM*	pET-Duet-TEV	*Nco*I
pCcaD-ET	*ccaD*	pET-Duet-TEV	*Nde*I
pCcaH-ET	*ccaH*	pET-Duet-TEV	*Nde*I
pCcaMD-ET	*ccaM*, *ccaD*	pET-Duet-TEV	*Nco*I, *Nde*I
pCcaMH-ET	*ccaM*, *ccaH*	pET-Duet-TEV	*Nco*I, *Nde*I
pCcaF-LIC	*ccaF*	pET-His6-TEV-LIC	*Ssp*I
pCcaM-LIC	*ccaM*	pET-His6-TEV-LIC	*Ssp*I
pCcaM$_N$-LIC	$ccaM_N(1\text{-}645)$	pET-His6-TEV-LIC	*Ssp*I
pCcaM$_C$-LIC	$ccaM_C(645\text{-}1015)$	pET-His6-TEV-LIC	*Ssp*I

Supplementary Table 2. Combination of plasmids for heterologous expression in *E. coli* BL21 (DE3).

Combination#	Enzymes	Antibiotics
pCcaA-RSF	His-tagged CcaA	kan
pCcaAM- RSF	His-tagged CcaA, CcaM	kan
pCcaA-RSF, pCcaBC-CDF	His-tagged CcaA, CcaB and CcaC	kan, spec
pCcaAD-RSF, pCcaBC-CDF	His-tagged CcaA, CcaB, CcaC and CcaD	kan, spec
pCcaAH-RSF, pCcaBC-CDF	His-tagged CcaA, CcaB, CcaC and CcaH	kan, spec
pCcaA(1-10)H-RSF, pCcaBC-CDF	His-tagged CcaA(1-10), CcaB, CcaC and CcaH	kan, spec
pCcaA(1-20)H-RSF, pCcaBC-CDF	His-tagged CcaA(1-20), CcaB, CcaC and CcaH	kan, spec
pCcaA(1-23)H-RSF, pCcaBC-CDF	His-tagged CcaA(1-23), CcaB, CcaC and CcaH	kan, spec
pCcaA(1-26)H-RSF, pCcaBC-CDF	His-tagged CcaA(1-26), CcaB, CcaC and CcaH	kan, spec
pCcaA(T7A)H-RSF, pCcaBC-CDF	His-tagged CcaA(T7A), CcaB, CcaC and CcaH	kan, spec
pCcaA(M1C)H-RSF, pCcaBC-CDF	His-tagged CcaA(M1C), CcaB, CcaC and CcaH	kan, spec
pCcaAH-RSF, pCcaBC-CDF, pCcaD-ET	His-tagged CcaA, CcaB, CcaC, CcaH and CcaD	kan, spec, amp
pCcaAD-RSF, pCcaBC-CDF, pCcaH-ET	His-tagged CcaA, CcaB, CcaC, CcaD and CcaH	kan, spec, amp
pCcaAD-RSF, pCcaBC-CDF, pCcaM-ET	His-tagged CcaA, CcaB, CcaC, CcaD and CcaM	kan, spec, amp
pCcaAD-RSF, pCcaBC-CDF, pCcaMH-ET	His-tagged CcaA, CcaB, CcaC, CcaD, CcaH and CcaM	kan, spec, amp
pCcaAH-RSF, pCcaBC-CDF, pCcaMD-ET	His-tagged CcaA, CcaB, CcaC, CcaH, CcaD and CcaM	kan, spec, amp

#Note: When the radical SAM protein CcaD was included in the co-expression system, pACYC-sufABCDSE (Chloramphenicol resistance) was also co-expressed to enhance the Fe-S cluster formation.

Supplementary Table 3. Primers used in this study.

Primer Name	Sequence (5' → 3')
CcaA-RSF1-FP	TACTTCCAAAGCCAGATGGAAAACCAAGCCGTA
CcaA-RSF1-RP	GCTCGAATTCGGATCTTAGCTGCAAGAATTTGT
CcaD-RSF2-FP	GAAGGAGATATACATATGCAAAAGAGCCTGAGT
CcaD-RSF2-RP	ATTGAGATCTGCCATTTAACATCCATAAAAGAAGCC
CcaM-RSF2-FP	GAAGGAGATATACATATGGATGTTAACGAAATA
CcaM-RSF2-RP	ATTGAGATCTGCCATTTATAGATTTTTGAAAGAATT
CcaH-RSF2-FP	GAAGGAGATATACATATGGATAACAGTGAAAAATTAACC
CcaH-RSF2-RP	ATTGAGATCTGCCATTTAAAGAGGCAGAGCGTT
CcaB-CDF1-FP	ATAAGGAGATATACCATGCGTATCCCGAAATTT
CcaB-CDF1-RP	ATGGCTGCTGCCCATTTAAATAAAGATGGAAATCGG
CcaC-CDF2-FP	GAAGGAGATATACATATGACCATGGAAAATAT
CcaC-CDF2-RP	ATTGAGATCTGCCATTTATAGATGGTTTCCTAAAAT
CcaM-ET1-FP	AGAAGGAGATATACCATGGATGTTAACGAAATA
CcaM-ET1-RP	ATGGCTGCTGCCCAT TTATAGATTTTTGAAAGAATT
CcaD-ET2-FP	GAAGGAGATATACATATGCAAAAGAGCCTGAGT
CcaD-ET2-FP	ATTGAGATCTGCCATTTAACATCCATAAAAGAAGCC
CcaH-ET2-FP	GAAGGAGATATACATATGGATAACAGTGAAAAATTAACC
CcaH-ET2-RP	ATTGAGATCTGCCATTTAAAGAGGCAGAGCGTT
CcaA-T7A-FP	TGCCAAAGCGCATCAAGTACCTGT
CcaA-T7A-RP	GGTACTTGATGCGCTTTGGCAGCT
CcaA-M1C-FP	CAAAGCCAGTGTGAAAACCAAGCC
CcaA-M1C -RP	TTGGTTTTCACACTGGCTTTGGAAGTA
CcaA-(1-10)-FP	TCAAGTACCTAAGTTACTGTACCA
CcaA-(1-10)-RP	TACAGTAACTTAGGTACTTGATGT
CcaA-(1-20)-FP	GTAAGTTGCTAATCAAGCGCATCT
CcaA-(1-20)-RP	TGCGCTTGATTAGCAACTTACACA
CcaA-(1-23)-FP	GTATCAAGCTAATCTACAAATTCT
CcaA-(1-23)-RP	ATTTGTAGATTAGCTTGATACGCA
CcaA-(1-26)-FP	GCATCTACATAATCTTGCAGCTAA
CcaA-(1-26)-RP	GCTGCAAGA TTA TGTAGATGCGCT
CcaF-His-FP	CTGTACTTCCAATCCAATGCAATGGCAGTTAAAATCGTTATT
CcaF-His-RP	CCGTTATCCACTTCCAATTTAGCTGCTTAAATTGATTCTG
CcaM-His-FP	CTGTACTTCCAATCCAATGCAATGGATGTTAACGAAATA
CcaM-His-RP	CCGTTATCCACTTCCAATTTATAGATTTTTGAAAGAATT
$CcaM_N$-His-FP	CTGTACTTCCAATCCAATGCAATGGATGTTAACGAAATA
$CcaM_N$-His-RP	CCGTTATCCACTTCCAATTTAATCCCCGCAATAGAAGGT
$CcaM_C$-His-FP	CTGTACTTCCAATCCAATGCAATGGATCATGAAGCACCTGTAGGC
$CcaM_C$-His-RP	CCGTTATCCACTTCCAATTTATAGATTTTTGAAAGAATT

Supplementary Table 4. ^{1}H chemical shifts of cCcaA^{BCH} in 9:1 (v/v) H_2O/ D_2O at 25 °C.

number	AA	NH	αH	βH	γH	δH	others
1	Ala		3.95	1.37			
2	A	8.51	4.29	1.29			
3	Ser	8.87	5.16	3.92			
4	thiazole				8.05		
5	Gln	8.62	4.48	2.1,1.99	2.29	NH_2: 7.37, 6.66	
6	Ser	8.78	5.03	3.86			
7	methyloxazole				2.36		
8	Ser	8.12	4.53	3.83			
9	Ser	8.69	5.04	3.86			
10	methyloxazole				2.36		
11	Cys-S-CH_3#	8.19	4.58	2.88, 2.78		1.96	
12	Val	8.37	4.17	2.02	0.75, 0.72		
13	Thr	N/A	4.10	5.21	1.21		
14	Val	8.50	4.38	1.94	0.80, 0.73		
15	Pro (*trans*)		4.36	2.17, 1.79	1.84, 1.80	3.64, 3.53	
16	Ser	8.72	5.14	3.91			
17	thiazole				8.06		
18	Val	8.23	4.24	2.03	0.81, 0.79		
19	Ser	8.85	5.08	3.88			
20	thiazole				8.045		
21	Val	8.27	4.29	2.08	0.82		
22	Ser	8.96	5.22	3.90			
23	oxazole				8.22		
24	Ala	8.30	4.41	1.33			
25	Ser	8.64	5.01	3.85			
26	methyloxazole				2.36		
27	Asn	8.35	4.80	2.75	NH_2: 7.48, 6.79		
28	Ser	8.81	5.21	3.91			
29	thiazole				8.041		
30	Ser	8.45	4.47	3.86			

^{1}H was referenced to H_2O at 4.80 ppm.

Observed STD peaks shown in green background (the indistinguishable proton of methyloxazole shown in teal background).

#: *S*-methylated Cys

Supplementary Table 5. ^{1}H chemical shifts of carnazolamide in CD_3OH at 25 °C.

Number	AA	NH	αH	βH	γH	δH
1	Ala		3.98	1.53		
2	Ala	8.728	4.82	1.465		
3	Dha	9.56		6.32, 5.583		
4	thiazole			8.24		
5	Gln	9.309	4.678	2.25	2.585, 2.459	7.60, 7.01 (NH_2)
6	Dha	9.58		6.18, 5.74		
7	methyloxazole			2.61		
8	Ser	8.15	4.78	3.99, 3.93		
9	Dha	9.53		6.20, 5.76		
10	methyloxazole			2.62		
11	Cys-S-CH_3[#]	8.04	4.68	2.93, 2.80		2.10
12	Val	8.24	4.03	1.90	0.73, 0.76	
13	Thr	N/A	4.78	4.05	0.784	
14	Val	8.646	4.608	2.05	0.90, 0.82	
15	Pro (*trans*)		4.84	2.50, 1.84	2.05	3.733, 3.652
16	Dha	10.55		6.30, 5.50		
17	thiazole					
18	Val	8.66	4.673	2.411	1.12	
19	Dha	10.39		5.89, 5.82		
20	thiazole			8.28		
21	Val	8.47	4.61	2.30	1.06	
22	Dha	9.68		6.17,5.83		
23	oxazole			8.46		
24	Ala	8.402	4.772	1.54		
25	Dha	9.42		6.164, 5.75		
26	methyloxazole					
27	Asn	8.378	5.127	2.95, 2.85	7.77, 7.22 (NH_2)	
28	Dha	10.15		6.26, 5.58		
29	thiazole					
30	Ser	8.94	4.74	4.03		

[#]: *S*-methylated Cys.

^{1}H was referenced to CD_3OH at 3.31 ppm.

Supplementary Table 6. Biological assay of carnazolamide.

Antibacterial Assay		
**Target strain**	**Gram**	**Carnazolamide#**
Escherichia coli ATC25592	-	No
Staphylococcus epidermidis 1457	+	No
Bacillus subtilis ATCC 6633	+	No
Micrococcus luteus	+	No
Lactococcus lactis	+	No
Staphylococcus carnosus TM300	+	No

Cytotoxicity Assay	
	**Carnazolamide***
VCaP cell line	> 20 μM
Caki-1 cell line	> 20 μM
MDA-MB-231 cell line	> 20 μM

#: Carnazolamide was dissolved in methanol as 1 mM stock solution, 5μl stock solution (5 nmol) was used to perform antibacterial activity assay with disc diffusion test, no antibacterial activity was observed.

*: Cytotoxicity assay was performed with the cell viability test using the alamarBlue assay method. No activities were observed with 20 μM top dose.

References.

1. Dong, S. H.; Tang, W.; Lukk, T.; Yu, Y.; Nair, S. K.; van der Donk, W. A. The enterococcal cytolysin synthetase has an unanticipated lipid kinase fold. *eLife* **4**, e07607 (2015).
2. Li, B.; Yu, J. P.; Brunzelle, J. S.; Moll, G. N.; van der Donk, W. A.; Nair, S. K. Structure and mechanism of the lantibiotic cyclase involved in nisin biosynthesis. *Science* **311**, 1464-1467 (2006).
3. Jumper, J. *et al.* Highly accurate protein structure prediction with AlphaFold. *Nature* **596**, 583-589 (2021).
4. Freeman, M. F. *et al.* Metagenome mining reveals polytheonamides as posttranslationally modified ribosomal peptides. *Science* **338**, 387–390 (2012).

Uncropped SDS-PAGE protein gel

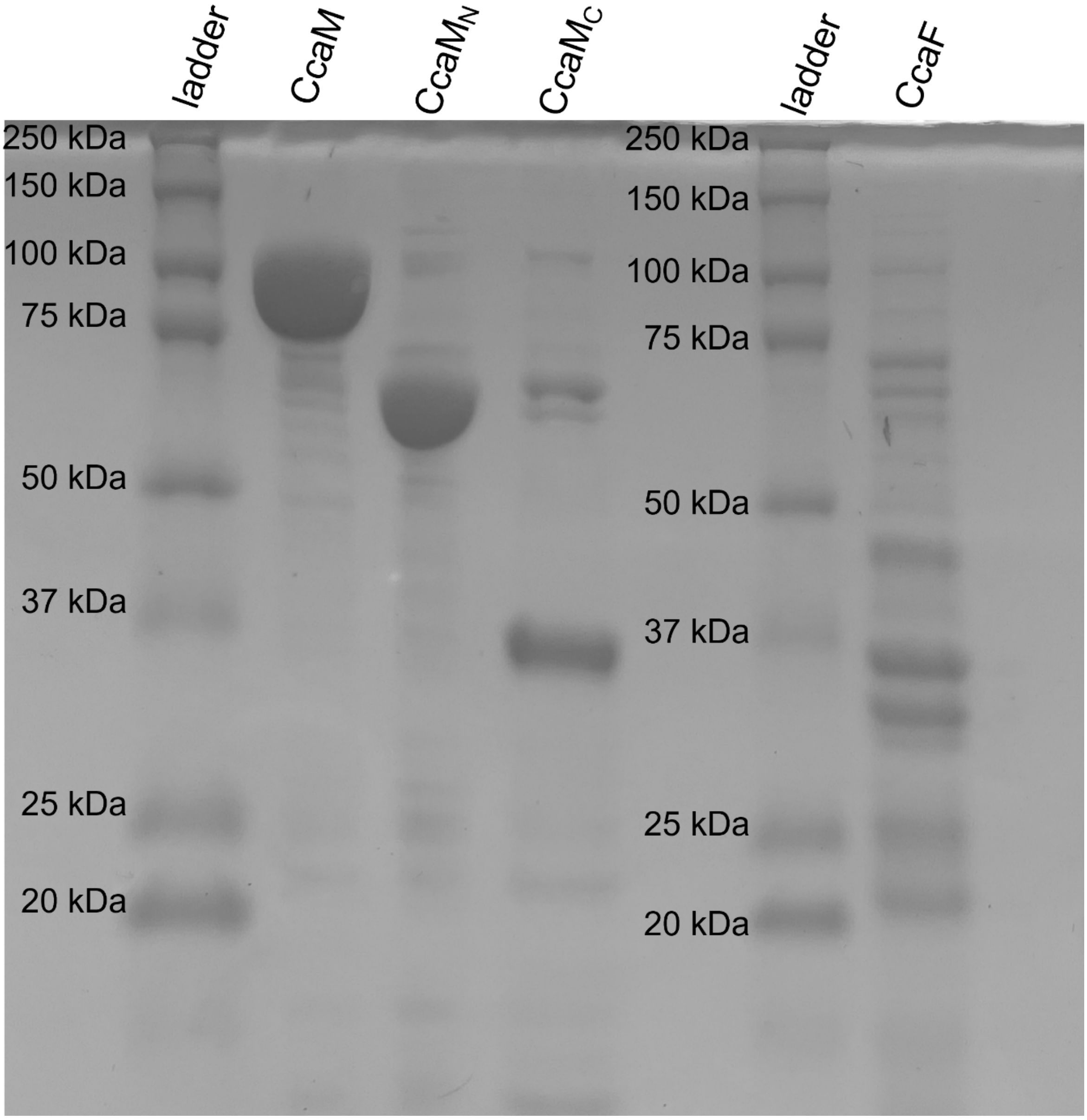

Supplement: Supplementary file 1 — Supplementary Information [file 41467_2023_43604_MOESM1_ESM.pdf]
